# Supplementary material for: Body‐Wide Inactivation of the Myc‐Like Mlx Transcription Factor Network Accelerates Aging and Increases the Lifetime Cancer Incidence
Source: Adv Sci (Weinh). 2024 Jul 8;11(34):2401593. doi: 10.1002/advs.202401593 (PMC11425880; doi:10.1002/advs.202401593)
Supplement: Supplementary file 1 — Supporting Information [file ADVS-11-2401593-s002.pdf]

ADVANCED SCIENCE
Open Access

Supporting Information

for *Adv. Sci.*, DOI 10.1002/advs.202401593

Body-Wide Inactivation of the Myc-Like Mlx Transcription Factor Network Accelerates Aging and Increases the Lifetime Cancer Incidence

*Huabo Wang, Taylor Stevens, Jie Lu, Alexander Roberts, Clinton Van't Land, Radhika Muzumdar, Zhenwei Gong, Jerry Vockley and Edward V. Prochownik**

Supporting Information for

Body-Wide Inactivation of the Myc-Like Mlx Transcription Factor Network Accelerates Aging and Increases the Lifetime Cancer incidence

Huabo Wang *et al.*

*Corresponding author: Edward V. Prochownik, Email: procev@chp.edu

This PDF file includes:

Tables S1 to S6
Figures S1 to S19

Other Supporting Information for this manuscript include the following:

Files S1 to S7

Table S1. Efficiency and Persistence of *Mlx* Gene Excision and Reduction of Expression Relative to Comparably Aged WT Tissues

Mouse ID	Age (months)	Tissue	qPCR (% knockout)	qRT-PCR (% expression vs. WT)
1	2	Liver	93	
2	2	Skeletal muscle	71	
3	2	Lung	80	
5	2	Kidney	77	
6	2	Stomach	82	
7	2	Small intestine	91	
8	2	Large intestine	94	
10	2	Pancreas	92	
11	2	Spleen	95	
12	2	Bone marrow	100	
14	2	Liver	89	
15	2	Skeletal muscle	89	
16	2	Lung	80	
17	2	Heart	73	
18	2	Kidney	79	
19	2	Stomach	85	
20	2	Small intestine	95	
21	2	Large intestine	95	
23	2	Pancreas	91	
24	2	Spleen	97	
25	2	Bone marrow	100	
52	5	Liver		2
53	5	Skeletal Muscle		26

54	5	Lung	21
55	5	Heart	30
56	5	Kidney	11
57	5	Stomach	8
58	5	Small Intestine	2
59	5	Large Intestine	0.6
60	5	Brain	51
61	5	Pancreas	10
62	5	Spleen	8
63	5	Bone Marrow	3
64	5	Adipose tissue	2
65	5	Liver	1
66	5	Skeletal Muscle	29
67	5	Lung	12
68	5	Heart	17
69	5	Kidney	7
70	5	Stomach	3
53	5	Small Intestine	0.2
54	5	Large Intestine	2
55	5	Brain	44
56	5	Pancreas	2
57	5	Spleen	5
58	5	Bone Marrow	2
59	5	Adipose tissue	1.5
4299	5	Skeletal Muscle	40
		Liver	1

		Adipose tissue		11
5001	5	Skeletal Muscle		23
		Liver		2
		Adipose tissue		5
5238	5	Skeletal Muscle		48
		Liver		1
		Adipose tissue		6
5239	5	Skeletal Muscle		30
		Liver		2
		Adipose tissue		9
27	5	Liver	84	
31	5	Kidney	86	
32	5	Stomach	71	
33	5	Small intestine	93	
34	5	Large intestine	99	
36	5	Pancreas	70	
37	5	Spleen	99	
38	5	Bone marrow	93	
42	5	Lung	79	
43	5	Heart	54	
46	5	Small Intestine	99	
47	5	Large Intestine	99	
49	5	Pancreas	58	
50	5	Spleen	99	
51	5	Bone Marrow	99	

7236	18	Adipose tissue		9
7237	18	Adipose tissue		10
7238	18	Adipose tissue		18
7239	18	Adipose tissue		15
7240	18	Adipose tissue		14
7116	18	Liver		0
7117	18	Liver		4
7118	18	Liver		4
7119	18	Liver		2
7174	18	Liver		2
7356	18	Skeletal muscle		61
7357	18	Skeletal muscle		44
7358	18	Skeletal muscle		41
7359	18	Skeletal muscle		30
7360	18	Skeletal muscle		52
5521	19	Bone marrow	95	
		Adipose tissue	89	
		Large intestine	88	
		Pancreas	78	
		Small intestine	91	
		Spleen	82	
5538	19	Adipose tissue	90	
		Liver	88	
5453	20	Bone marrow	91	

		Adipose tissue	86	
		Heart	74	
		Liver	93	
3868	21	Heart	82	
		Kidney	89	
		Large intestine	91	
		Pancreas	80	
		Small intestine	83	
		Spleen	89	
		Stomach	83	
5311	22	Adipose tissue	89	
		Liver	85	
		Spleen	85	
5593	25	Small intestine	89	
		Liver	92	
		Large intestine	88	
		Spleen	89	
		Kidney	61	
5638	27	Spleen	98	
5433	31	Liver	82	
		Stomach	94	
		Kidney	79	
		Spleen	95	
		Large Intestine	85	
5532	31	Liver	96	
		Pancreas	64	
		Stomach	90	

	Small Intestine	91	
	Kidney	70	
	Large intestine	90	

Table S2. Serum acylcarnitine levels: 5 month WT and MlxKO mice

	P value	Mean of WT 5mos	Mean of MlxKO 5 mos	Difference	SE of difference	t ratio
C0	0.548594	34.71	38.75	-4.043	6.416	0.6302
C2	0.549941	54.89	48.82	6.064	9.655	0.628
C3	0.933289	1.936	1.984	-0.04755	0.548	0.08676
C4	0.427705	1.823	1.375	0.4478	0.5319	0.8418
C5	0.949985	0.2986	0.2933	0.00535	0.0823	0.06501
C6	0.845501	0.2048	0.2145	-0.0097	0.04797	0.2022
C7	0.660826	0.1328	0.1428	-0.00995	0.02172	0.458
C8	0.833693	0.113	0.1183	-0.00525	0.02409	0.2179
C9	0.281512	0.0712	0.08725	-0.01605	0.01376	1.167
C10	0.064004	0.1304	0.2163	-0.08585	0.03907	2.197
C12	0.637483	0.1066	0.1188	-0.01215	0.02467	0.4924
C14	0.276868	0.2424	0.298	-0.0556	0.04715	1.179
C16	0.74747	0.652	0.6203	0.03175	0.09479	0.335
C18	0.699576	0.2104	0.1938	0.01665	0.0414	0.4021
C8:1	0.289567	0.1066	0.1888	-0.08215	0.0717	1.146
C10:1	0.236804	0.1496	0.2053	-0.05565	0.04301	1.294
C3:1	0.393799	0.083	0.1118	-0.02875	0.03164	0.9085
Crotonyl / Me	0.435421	0.1394	0.2008	-0.06135	0.07417	0.8272
Figlu	0.995623	0.1574	0.1573	0.00015	0.02639	0.005685
C5:1	0.342821	0.0608	0.08175	-0.02095	0.02059	1.017
C3-OH	0.435213	0.3656	0.3328	0.03285	0.03969	0.8276
C4-OH	0.311007	0.7836	0.8593	-0.07565	0.06928	1.092
C5-OH	0.441613	0.1724	0.1898	-0.01735	0.02127	0.8156
C6-OH	0.262673	0.0924	0.117	-0.0246	0.0202	1.218
C2DC	0.492812	0.0632	0.07675	-0.01355	0.01873	0.7235
C8-OH	0.606769	0.1462	0.154	-0.0078	0.01448	0.5387
C10:2	0.352861	0.0828	0.1625	-0.0797	0.0801	0.995
C4DC	0.342363	0.1168	0.156	-0.0392	0.03849	1.018
C10:1-OH	0.599678	0.1106	0.0965	0.0141	0.02565	0.5496
C10-OH (C5D	0.699714	0.1208	0.1328	-0.01195	0.02973	0.4019
C12:1	0.864531	0.0876	0.09125	-0.00365	0.02062	0.177
C6DC	0.690628	0.426	0.39	0.036	0.08677	0.4149
C12:1-OH	0.264378	0.0722	0.149	-0.0768	0.0633	1.213
C12-OH	0.545436	0.1208	0.099	0.0218	0.03431	0.6353
C14:2	0.00927	0.2566	0.1153	0.1414	0.03975	3.556
C14:1	0.798863	0.1952	0.1865	0.0087	0.03287	0.2647
C8DC	0.184811	0.1434	0.2098	-0.06635	0.04511	1.471
C14:1-OH	0.082436	0.0806	0.1268	-0.04615	0.02278	2.026
C14-OH	0.578705	0.0514	0.05975	-0.00835	0.01434	0.5822
C16:2	0.559678	0.12	0.131	-0.011	0.01796	0.6123
C16:1	0.89597	0.229	0.2345	-0.0055	0.04057	0.1356
C10DC	0.172962	0.072	0.09425	-0.02225	0.01466	1.517
C16-OH	0.187956	0.0854	0.1083	-0.02285	0.01566	1.459
C16:1-OH	0.187956	0.0854	0.1083	-0.02285	0.01566	1.459
C18:3	0.012975	0.1778	0.055	0.1228	0.03712	3.308
C18:2	0.813765	0.26	0.2745	-0.0145	0.05928	0.2446
C18:1	0.392628	0.6884	0.536	0.1524	0.1673	0.9109
C12DC	0.057416	0.0538	0.078	-0.0242	0.01066	2.271
C18:2-OH	0.635748	0.078	0.06825	0.00975	0.0197	0.495
C18:1-OH	0.331346	0.0978	0.0785	0.0193	0.01849	1.044
C18-OH	0.41041	0.063	0.07775	-0.01475	0.01685	0.8754

Table S3. Serum acylcarnitine levels: 20 month WT and *Mlx* KO mice

	P value	Mean of WT 20mos	Mean of MlxKO 20mos	Difference	SE of difference	t ratio
C0	0.625899	43.63	41.14	2.489	4.931	0.5047
C2	0.888484	53.57	52.7	0.8707	6.036	0.1442
C3	0.796305	1.505	1.423	0.0819	0.308	0.2659
C4	0.074544	2.666	1.615	1.05	0.5208	2.016
C5	0.869218	0.1652	0.171	-0.005833	0.03443	0.1694
C6	0.050208	0.338	0.2328	0.1052	0.04656	2.26
C7	0.994275	0.1333	0.1334	-6.67E-05	0.009037	0.007377
C8	0.718718	0.1138	0.1174	-0.003567	0.009596	0.3717
C9	0.528966	0.116	0.1216	-0.0056	0.008552	0.6548
C10	0.798725	0.1375	0.144	-0.0065	0.02475	0.2627
C12	0.956378	0.1052	0.106	-0.0008333	0.01482	0.05624
C14	0.565468	0.1833	0.203	-0.01967	0.03296	0.5966
C16	0.852866	0.4013	0.389	0.01233	0.06462	0.1909
C18	0.135671	0.1325	0.1094	0.0231	0.0141	1.639
C8:1	0.358068	0.5018	0.3408	0.161	0.1663	0.9686
C10:1	0.261019	0.3303	0.223	0.1073	0.08949	1.199
C3:1	0.228638	0.2602	0.1662	0.09397	0.07275	1.292
Crotonyl / Me-Acrylyl	0.37111	0.5775	0.3714	0.2061	0.2189	0.9413
Figlu	0.349148	0.321	0.2072	0.1138	0.1152	0.9876
C5:1	0.045734	0.042	0.0538	-0.0118	0.005093	2.317
C3-OH	0.109183	0.2785	0.2094	0.0691	0.03887	1.778
C4-OH	0.188395	1.662	1.146	0.5164	0.3628	1.423
C5-OH	0.289939	0.1005	0.1184	-0.0179	0.01592	1.124
C6-OH	0.96035	0.1013	0.1022	-0.0008667	0.01696	0.05112
C2DC	0.94062	0.193	0.1908	0.0022	0.02872	0.0766
C8-OH	0.292902	0.3257	0.1532	0.1725	0.1544	1.117
C10:2	0.16925	0.2447	0.1532	0.09147	0.0612	1.495
C4DC	0.148943	0.3838	0.2674	0.1164	0.07377	1.578
C10:1-OH	0.405108	0.1365	0.1086	0.0279	0.03194	0.8735
C10-OH (C5DC)	0.469388	0.1117	0.1222	-0.01053	0.01395	0.7553
C12:1	0.846614	0.08933	0.0874	0.001933	0.009711	0.1991
C6DC	0.921003	0.2507	0.2538	-0.003133	0.03072	0.102
C12:1-OH	0.828888	0.063	0.0646	-0.0016	0.007191	0.2225
C12-OH	0.182724	0.07483	0.0622	0.01263	0.008751	1.444
C14:2	0.475765	0.1437	0.136	0.007667	0.0103	0.7441
C14:1	0.492798	0.2193	0.2028	0.01653	0.02313	0.7149
C8DC	0.294664	0.06017	0.0798	-0.01963	0.01764	1.113
C14:1-OH	0.854228	0.08083	0.0832	-0.002367	0.01252	0.1891
C14-OH	0.343857	0.07433	0.0874	-0.01307	0.01308	0.9991
C16:2	0.373093	0.07133	0.0868	-0.01547	0.0165	0.9373
C16:1	0.831389	0.2312	0.238	-0.006833	0.03117	0.2192
C10DC	0.915181	0.4188	0.4158	0.003033	0.02769	0.1095
C16-OH	0.099289	0.1192	0.0804	0.03877	0.0211	1.838
C16:1-OH	0.126941	0.08233	0.0958	-0.01347	0.008008	1.682
C18:3	0.414354	0.058	0.0524	0.0056	0.006544	0.8557
C18:2	0.194067	0.1787	0.2058	-0.02713	0.01934	1.403
C18:1	0.288622	0.526	0.4694	0.0566	0.05019	1.128
C12DC	0.710472	0.3005	0.3076	-0.0071	0.01853	0.3832
C18:2-OH	0.629184	0.06733	0.0716	-0.004267	0.008536	0.4998
C18:1-OH	0.560507	0.08667	0.0792	0.007467	0.01235	0.6044
C18-OH	0.002285	0.0645	0.0388	0.0257	0.00611	4.206

Table S4. Serum acylcarnitine levels: 5 month vs. 20 month WT mice

	P value	Mean of WT 20mos	Mean of WT 5mos	Difference	SE of difference	t ratio
C0	0.137324	43.63	34.71	8.923	5.471	1.631
C2	0.867123	53.57	54.89	-1.316	7.645	0.1722
C3	0.338557	1.505	1.936	-0.4317	0.4271	1.011
C4	0.198952	2.666	1.823	0.8429	0.6079	1.387
C5	0.054415	0.1652	0.2986	-0.1334	0.06037	2.21
C6	0.045689	0.338	0.2048	0.1332	0.05748	2.317
C7	0.963978	0.1333	0.1328	0.0005333	0.01149	0.04643
C8	0.962316	0.1138	0.113	0.0008333	0.01715	0.04858
C9	0.000936	0.116	0.0712	0.0448	0.009278	4.829
C10	0.696317	0.1375	0.1304	0.0071	0.01762	0.4031
C12	0.940283	0.1052	0.1066	-0.001433	0.01861	0.07703
C14	0.134662	0.1833	0.2424	-0.05907	0.03594	1.644
C16	0.006241	0.4013	0.652	-0.2507	0.07066	3.547
C18	0.041676	0.1325	0.2104	-0.0779	0.03282	2.373
C8:1	0.040676	0.5018	0.1066	0.3952	0.1655	2.388
C10:1	0.076358	0.3303	0.1496	0.1807	0.0903	2.002
C3:1	0.039652	0.2602	0.083	0.1772	0.0737	2.404
Crotonyl / Me-Acrylyl	0.071318	0.5775	0.1394	0.4381	0.2143	2.044
Figlu	0.187693	0.321	0.1574	0.1636	0.1147	1.426
C5:1	0.022373	0.042	0.0608	-0.0188	0.006829	2.753
C3-OH	0.093491	0.2785	0.3656	-0.0871	0.04644	1.875
C4-OH	0.023059	1.662	0.7836	0.8786	0.3213	2.734
C5-OH	0.00117	0.1005	0.1724	-0.0719	0.0154	4.669
C6-OH	0.614363	0.1013	0.0924	0.008933	0.01712	0.5219
C2DC	0.00004	0.193	0.0632	0.1298	0.01746	7.433
C8-OH	0.275325	0.3257	0.1462	0.1795	0.1545	1.161
C10:2	0.02499	0.2447	0.0828	0.1619	0.06028	2.685
C4DC	0.001343	0.3838	0.1168	0.267	0.05841	4.572
C10:1-OH	0.452006	0.1365	0.1106	0.0259	0.03295	0.7861
C10-OH (C5DC)	0.675613	0.1117	0.1208	-0.009133	0.02112	0.4324
C12:1	0.846046	0.08933	0.0876	0.001733	0.008673	0.1998
C6DC	0.002847	0.2507	0.426	-0.1753	0.0432	4.059
C12:1-OH	0.12923	0.063	0.0722	-0.0092	0.005509	1.67
C12-OH	0.097276	0.07483	0.1208	-0.04597	0.02484	1.85
C14:2	0.007265	0.1437	0.2566	-0.1129	0.03273	3.451
C14:1	0.270265	0.2193	0.1952	0.02413	0.02054	1.175
C8DC	0.008132	0.06017	0.1434	-0.08323	0.02463	3.38
C14:1-OH	0.98218	0.08083	0.0806	0.0002333	0.01016	0.02296
C14-OH	0.066759	0.07433	0.0514	0.02293	0.011	2.085
C16:2	0.002912	0.07133	0.12	-0.04867	0.01204	4.044
C16:1	0.95346	0.2312	0.229	0.002167	0.03611	0.06001
C10DC	<0.000001	0.4188	0.072	0.3468	0.01715	20.22
C16-OH	0.169558	0.1192	0.0854	0.03377	0.02261	1.493
C16:1-OH	0.791838	0.08233	0.0854	-0.003067	0.01128	0.2719
C18:3	0.00276	0.058	0.1778	-0.1198	0.02937	4.08
C18:2	0.021146	0.1787	0.26	-0.08133	0.02918	2.787
C18:1	0.211098	0.526	0.6884	-0.1624	0.1206	1.346
C12DC	<0.000001	0.3005	0.0538	0.2467	0.01729	14.27
C18:2-OH	0.442574	0.06733	0.078	-0.01067	0.01328	0.8032
C18:1-OH	0.503325	0.08667	0.0978	-0.01113	0.01597	0.6971
C18-OH	0.859542	0.0645	0.063	0.0015	0.008237	0.1821

Table S5. Serum acylcarnitine levels: 5 month vs. 20 month *Mlx* KO mice

	P value	Mean of MlxKO 20mos	Mean of MlxKO 5 mos	Difference	SE of difference	t ratio
C0	0.686521	41.14	38.75	2.392	5.683	0.4208
C2	0.627807	52.7	48.82	3.877	7.648	0.5069
C3	0.206123	1.423	1.984	-0.5612	0.4027	1.393
C4	0.523088	1.615	1.375	0.2404	0.3577	0.6721
C5	0.05724	0.171	0.2933	-0.1223	0.05379	2.273
C6	0.443384	0.2328	0.2145	0.0183	0.02253	0.8123
C7	0.651386	0.1334	0.1428	-0.00935	0.01982	0.4719
C8	0.959529	0.1174	0.1183	-0.00085	0.01616	0.05259
C9	0.03324	0.1216	0.08725	0.03435	0.01299	2.644
C10	0.150525	0.144	0.2163	-0.07225	0.04476	1.614
C12	0.548378	0.106	0.1188	-0.01275	0.02022	0.6305
C14	0.06571	0.203	0.298	-0.095	0.04359	2.179
C16	0.033604	0.389	0.6203	-0.2313	0.08772	2.636
C18	0.002319	0.1094	0.1938	-0.08435	0.01811	4.658
C8:1	0.080433	0.3408	0.1888	0.1521	0.07445	2.042
C10:1	0.672792	0.223	0.2053	0.01775	0.04029	0.4406
C3:1	0.092199	0.1662	0.1118	0.05445	0.02793	1.95
Crotonyl / Me-Acrylyl	0.10904	0.3714	0.2008	0.1707	0.09296	1.836
Figlu	0.134249	0.2072	0.1573	0.04995	0.0295	1.693
C5:1	0.200578	0.0538	0.08175	-0.02795	0.01978	1.413
C3-OH	0.001213	0.2094	0.3328	-0.1234	0.02359	5.23
C4-OH	0.23915	1.146	0.8593	0.2866	0.2227	1.287
C5-OH	0.013816	0.1184	0.1898	-0.07135	0.02187	3.263
C6-OH	0.482919	0.1022	0.117	-0.0148	0.01998	0.7408
C2DC	0.012548	0.1908	0.07675	0.1141	0.03422	3.332
C8-OH	0.94974	0.1532	0.154	-0.0008	0.01225	0.06533
C10:2	0.912024	0.1532	0.1625	-0.0093	0.08119	0.1145
C4DC	0.147661	0.2674	0.156	0.1114	0.06845	1.627
C10:1-OH	0.623387	0.1086	0.0965	0.0121	0.02356	0.5135
C10-OH (C5DC)	0.647129	0.1222	0.1328	-0.01055	0.02207	0.4781
C12:1	0.861941	0.0874	0.09125	-0.00385	0.02134	0.1804
C6DC	0.124009	0.2538	0.39	-0.1362	0.07793	1.748
C12:1-OH	0.225922	0.0646	0.149	-0.0844	0.06357	1.328
C12-OH	0.080444	0.0622	0.099	-0.0368	0.01802	2.042
C14:2	0.029216	0.136	0.1153	0.02075	0.007593	2.733
C14:1	0.659784	0.2028	0.1865	0.0163	0.03547	0.4595
C8DC	0.013478	0.0798	0.2098	-0.13	0.03961	3.28
C14:1-OH	0.119517	0.0832	0.1268	-0.04355	0.02456	1.773
C14-OH	0.145256	0.0874	0.05975	0.02765	0.01687	1.639
C16:2	0.09476	0.0868	0.131	-0.0442	0.02289	1.931
C16:1	0.919716	0.238	0.2345	0.0035	0.0335	0.1045
C10DC	0.000017	0.4158	0.09425	0.3216	0.031	10.37
C16-OH	0.051298	0.0804	0.1083	-0.02785	0.01187	2.347
C16:1-OH	0.336763	0.0958	0.1083	-0.01245	0.01207	1.031
C18:3	0.785854	0.0524	0.055	-0.0026	0.009209	0.2823
C18:2	0.232281	0.2058	0.2745	-0.0687	0.05253	1.308
C18:1	0.505545	0.4694	0.536	-0.0666	0.09492	0.7017
C12DC	<0.000001	0.3076	0.078	0.2296	0.01355	16.95
C18:2-OH	0.829509	0.0716	0.06825	0.00335	0.01499	0.2235
C18:1-OH	0.959889	0.0792	0.0785	0.0007	0.01343	0.05212
C18-OH	0.038853	0.0388	0.07775	-0.03895	0.01535	2.537

Table S6. Antibodies used in the current study.

Name of Antibody	Vendor	Catalog no.	Dilution used
GAPDH	Sigma	G8795	1:10000
GLUT1	Abcam	AB115730	1:2000
GLUT2	ProteinTech	20436-1-AP	1:300
GLUT4	Cell Signaling Technology	2213	1:1000
PDH	Cell Signaling Technology	3205	1:1000
PFKL	Aviva System Biology	ARP45774	1:1000
PFKM	R&D Systems	MAB7687	1:3000
PGC1	Novus Biological	NBP1-04676	1:1000
pPDH-E1α(pSer293)	Millipore (Calbiochem)	AP1062	1:1000

Fig. S1

A

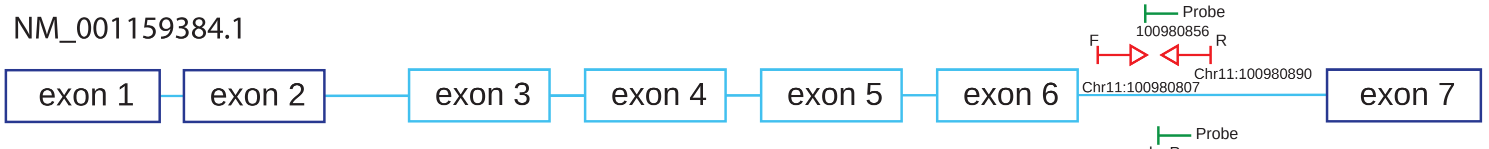

B

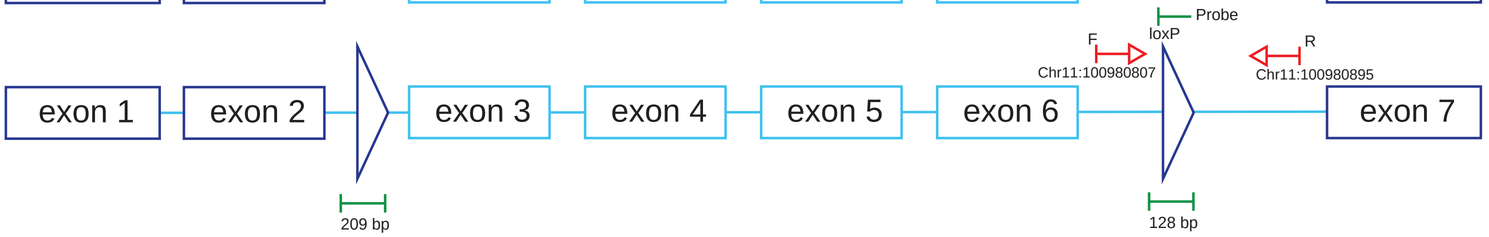

C

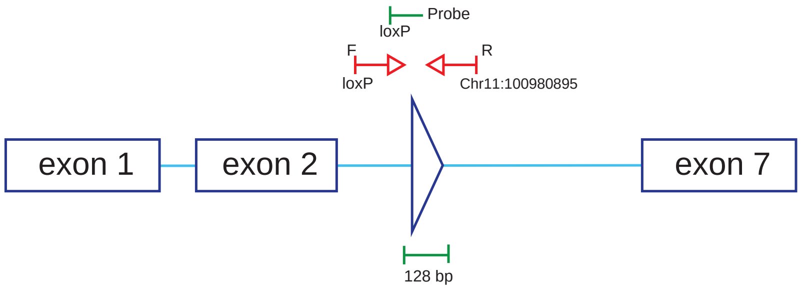

D

		Start bp	End bp	Sequence(5'-3')
MLX EXON 1 (NC_000077.7)	Fwd	100978116	100978132	AGTCCGCTGGCTTGTTT
	Rev	100978192	100978176	TTGACCCAAGGGTCCTC
	Probe	100978134	100978157	/56-FAM/CGGTTCGGT/ZEN/AGGTTCACGATGACG/3IABkFQ/
MLX EXON 7 (NC_000077.7)	Fwd	100980596	100980617	CTCCCTCTTCCAGTCTTTCAAC
	Rev	100980691	100980672	GGCTCACAGTGTTCCTCAAT
	Probe	100980655	100980669	/56-FAM/AGCTCTCAG/ZEN/CTTGTGTCTTCAGCT/3IABkFQ/
WT (NC_000077.7)	Fwd	100980807	100980825	TAGCCCAGTGAAGGTCTCA
	Rev	100980911	100980890	AGGAGTAGACAGGGTAGCTAAT
	Probe	100980856	100980879	/56-FAM/CAGGTCCAGCTTTAGCCCATGTCA/3IABkFQ/
mlx loxP/loxP (NC_000077.7)	Fwd	100980807	100980825	TAGCCCAGTGAAGGTCTCA
	Rev	100980914	100980895	CCAAGGAGTAGACAGGGTAGAA
	Probe	loxP	loxP	/56-FAM/ACTAGTTCTAGAGCGGCCAATTCGC/3IABkFQ/
mlx -/- (KO) (NC_000077.7)	Fwd	loxP	loxP	CAGGTCGAGGGACCTAATAACT
	Rev	100980914	100980895	CCAAGGAGTAGACAGGGTAGAA
	Probe	loxP	loxP	/56-FAM/ACTAGTTCTAGAGCGGCCAATTCGC/3IABkFQ/

E

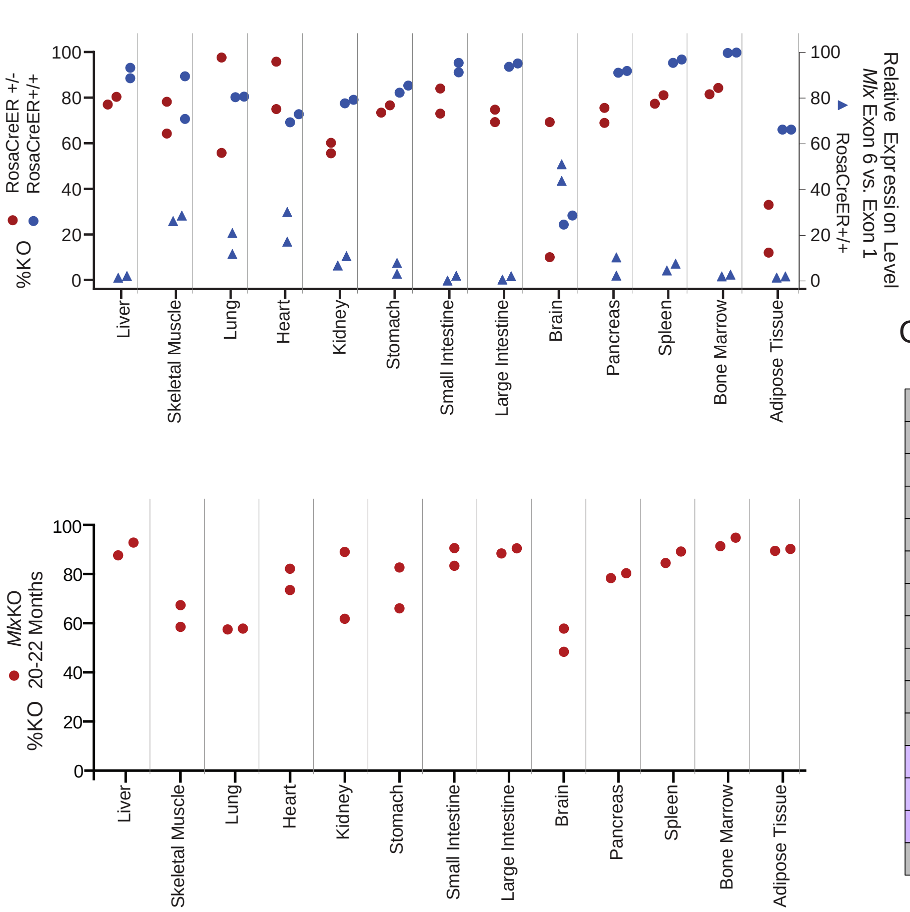

F

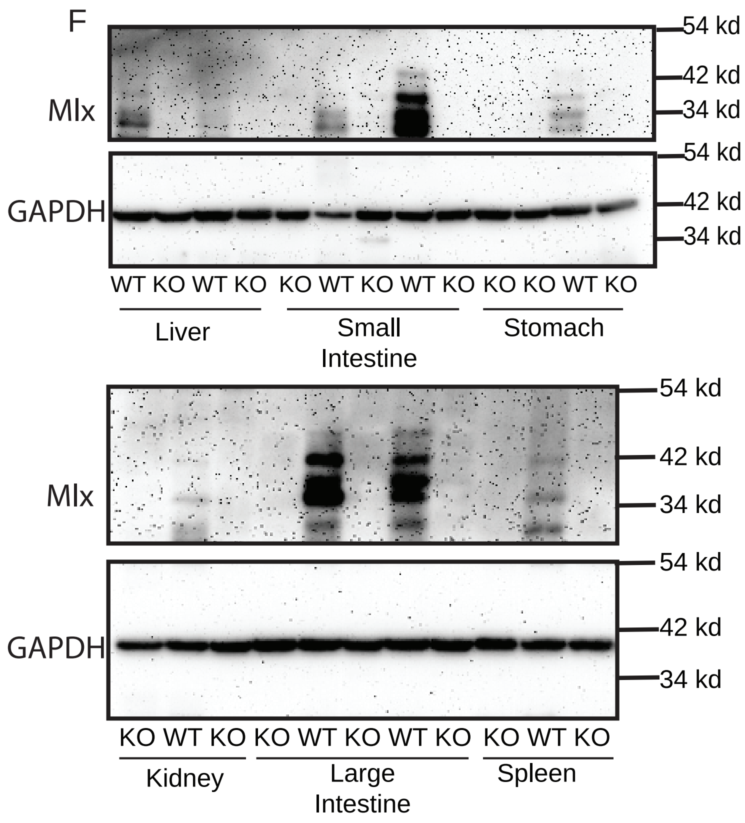

G

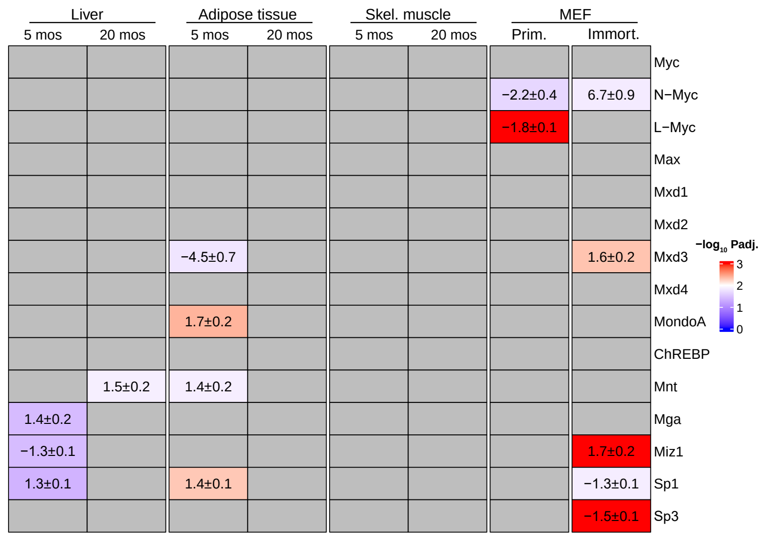

Figure S1. Strategy for Taq-Man-based qPCR and qRT-PCR assays for quantifying "floxed" (WT) and excised *Mlx* loci.

(A) The WT *Mlx* locus and the unexcised $Mlx^{loxP/loxP}$ locus.

(B) LoxP sites flanking exons 3 and 6 are indicated by the red triangles. Red arrows in A indicate the primer set used for identifying WT alleles. The TaqMan probe is shown in green. Numbering of primers and probes is based on their correspondence to the sequence of the Mlx genomic locus (NC_000077.7 (100977538..100983033)). Black arrows indicate the primer set used to uniquely identify $Mlx^{loxP/loxP}$ alleles. The unique TaqMan probe used to quantify the PCR products is depicted in green.

(C) The *Mlx*KO locus following Cre-recombinase mediated recombination. Blue arrows indicate primers used to amplify KO (*Mlx*-/-) alleles.

(D) Sequences, genomic locations, and dyes for primers and probes shown in **A**-**C** and for quantification of WT transcripts by qRT-PCR.

(E) Excisional efficiency of the *Mlx* locus in tissues of randomly chosen *Mlx*KO mice 2 months after tamoxifen treatment. The indicated tissues were sampled from mice bearing either one or 2 copies of the CreER transgene. qRT-PCR results in each *Mlx*KO tissue were compared to the results obtained in the identical tissues from WT mice.

(F) Absence of Mlx protein in *Mlx*KO tissues. Total cell lysates from the indicated, age-matched WT and *Myc*KO tissues were examined by immuno-blotting for the expression of *Mlx*. GAPDH was used as a loading control.

(G). Differential transcript levels for all members of the Extended Myc Network in liver, adipose tissue and skeletal muscle from mice of the indicated ages and from primary and immortalized MEFs[7f]. Actual fold-differences (+/- 1S.E.) between *Mlx*KO versus WT tissues are indicated within individual colored boxes. For all tissues, N=5 samples/group. For cell lines N=3 samples/group.

Supp. fig 2

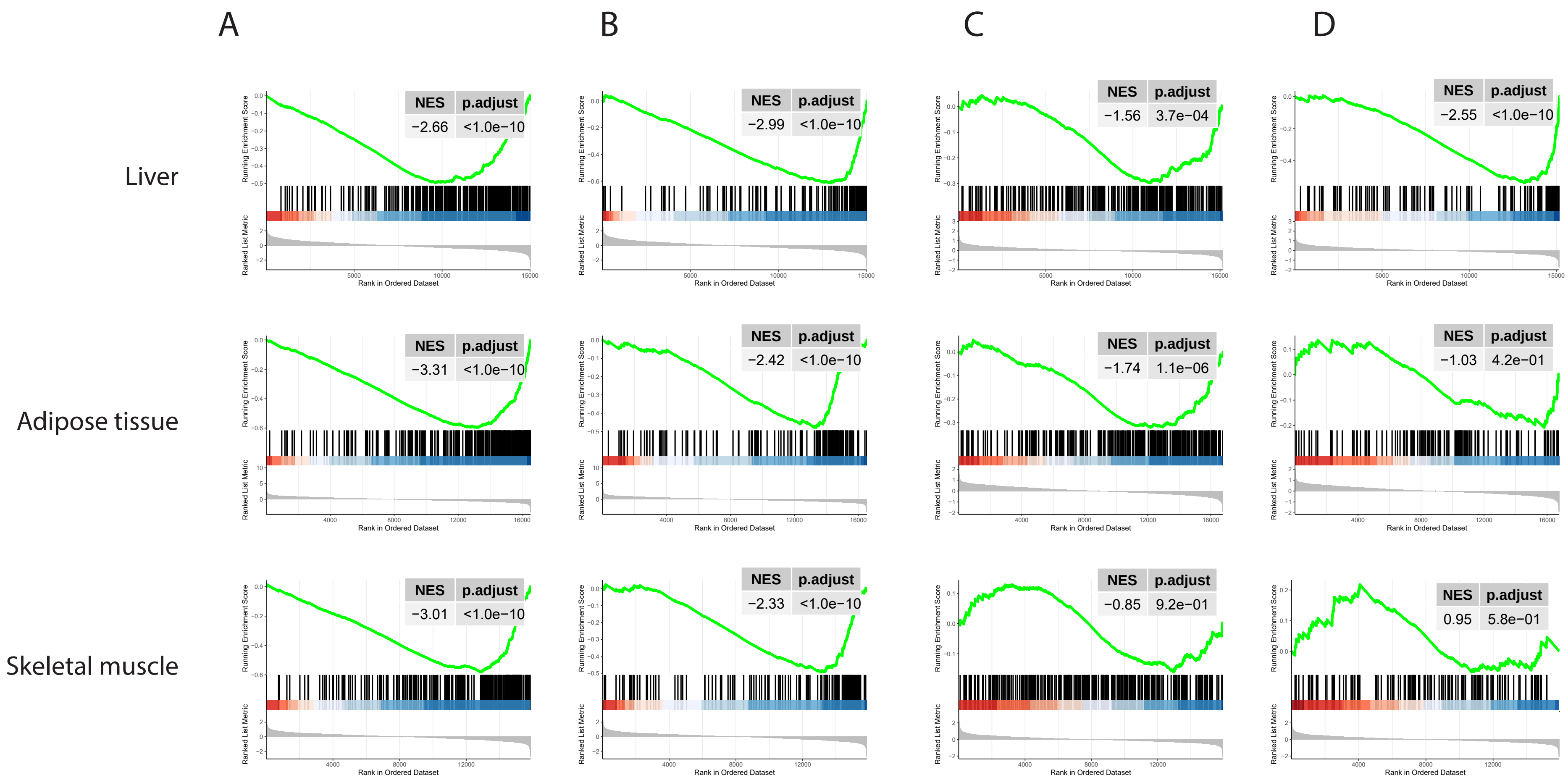
A
B
C
D
Liver
Adipose tissue
Skeletal muscle
NES p.adjust
−2.66 <1.0e−10
−2.99 <1.0e−10
−1.56 3.7e−04
−2.55 <1.0e−10
−3.31 <1.0e−10
−2.42 <1.0e−10
−1.74 1.1e−06
−1.03 4.2e−01
−3.01 <1.0e−10
−2.33 <1.0e−10
−0.85 9.2e−01
0.95 5.8e−01
Running Enrichment Score
Ranked List Metric
Rank in Ordered Dataset

Figure S2. GSEA with validated ChREBP, MondoA and/or Mlx target genes

(A). GSEA of 264 ChREBP/MondoA/Mlx target genes from the Enrichr datbase gene set ARCHS4 in the indicated tissues of 5 month old *Mlx*KO versus WT mice.

(B). GSEA of a 181 member ChREBP/MondoA/Mlx direct target gene set from the Qiagen database in the indicated tissues of 5 month old *Mlx*KO versus WT mice.

(C). GSEA of the gene set from **A** performed on tissues from 5 month old *Myc*KO mice.

(D). GSEA of the gene set from **B** performed on tissues from 5 month old *Myc*KO mice.

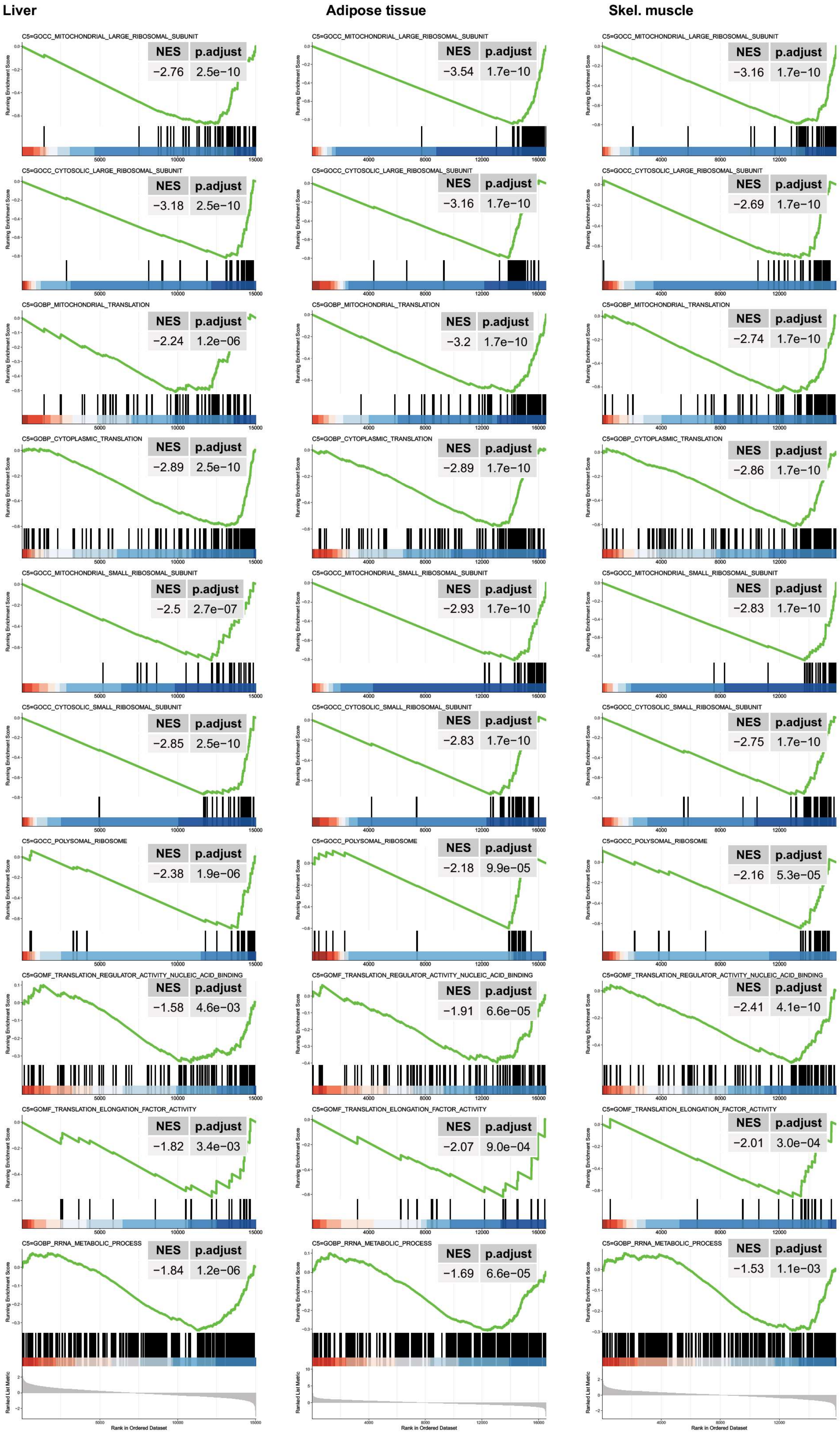
Liver
Adipose tissue
Skel. muscle
C5=GOCC_MITOCHONDRIAL_LARGE_RIBOSOMAL_SUBUNIT
NES p.adjust
−2.76 2.5e−10
−3.54 1.7e−10
−3.16 1.7e−10
C5=GOCC_CYTOSOLIC_LARGE_RIBOSOMAL_SUBUNIT
−3.18 2.5e−10
−3.16 1.7e−10
−2.69 1.7e−10
C5=GOBP_MITOCHONDRIAL_TRANSLATION
−2.24 1.2e−06
−3.2 1.7e−10
−2.74 1.7e−10
C5=GOBP_CYTOPLASMIC_TRANSLATION
−2.89 2.5e−10
−2.89 1.7e−10
−2.86 1.7e−10
C5=GOCC_MITOCHONDRIAL_SMALL_RIBOSOMAL_SUBUNIT
−2.5 2.7e−07
−2.93 1.7e−10
−2.83 1.7e−10
C5=GOCC_CYTOSOLIC_SMALL_RIBOSOMAL_SUBUNIT
−2.85 2.5e−10
−2.83 1.7e−10
−2.75 1.7e−10
C5=GOCC_POLYSOMAL_RIBOSOME
−2.38 1.9e−06
−2.18 9.9e−05
−2.16 5.3e−05
C5=GOMF_TRANSLATION_REGULATOR_ACTIVITY_NUCLEIC_ACID_BINDING
−1.58 4.6e−03
−1.91 6.6e−05
−2.41 4.1e−10
C5=GOMF_TRANSLATION_ELONGATION_FACTOR_ACTIVITY
−1.82 3.4e−03
−2.07 9.0e−04
−2.01 3.0e−04
C5=GOBP_RRNA_METABOLIC_PROCESS
−1.84 1.2e−06
−1.69 6.6e−05
−1.53 1.1e−03
Running Enrichment Score
Ranked List Metric
Rank in Ordered Dataset

Figure S3. "Translation/ribosomal structure & function" GSEA profiles in liver, adipose tissue and skeletal muscle from 5 month old *Mlx*KO mice relative to WT controls

Normalized enrichment scores and q values are indicated in the upper right corner of each profile. Data used to generate the ridge plots shown in Figure 6A are re-graphed and included here along with additional representative profiles. See File S1 for a complete list of all gene sets of significance included in this category.

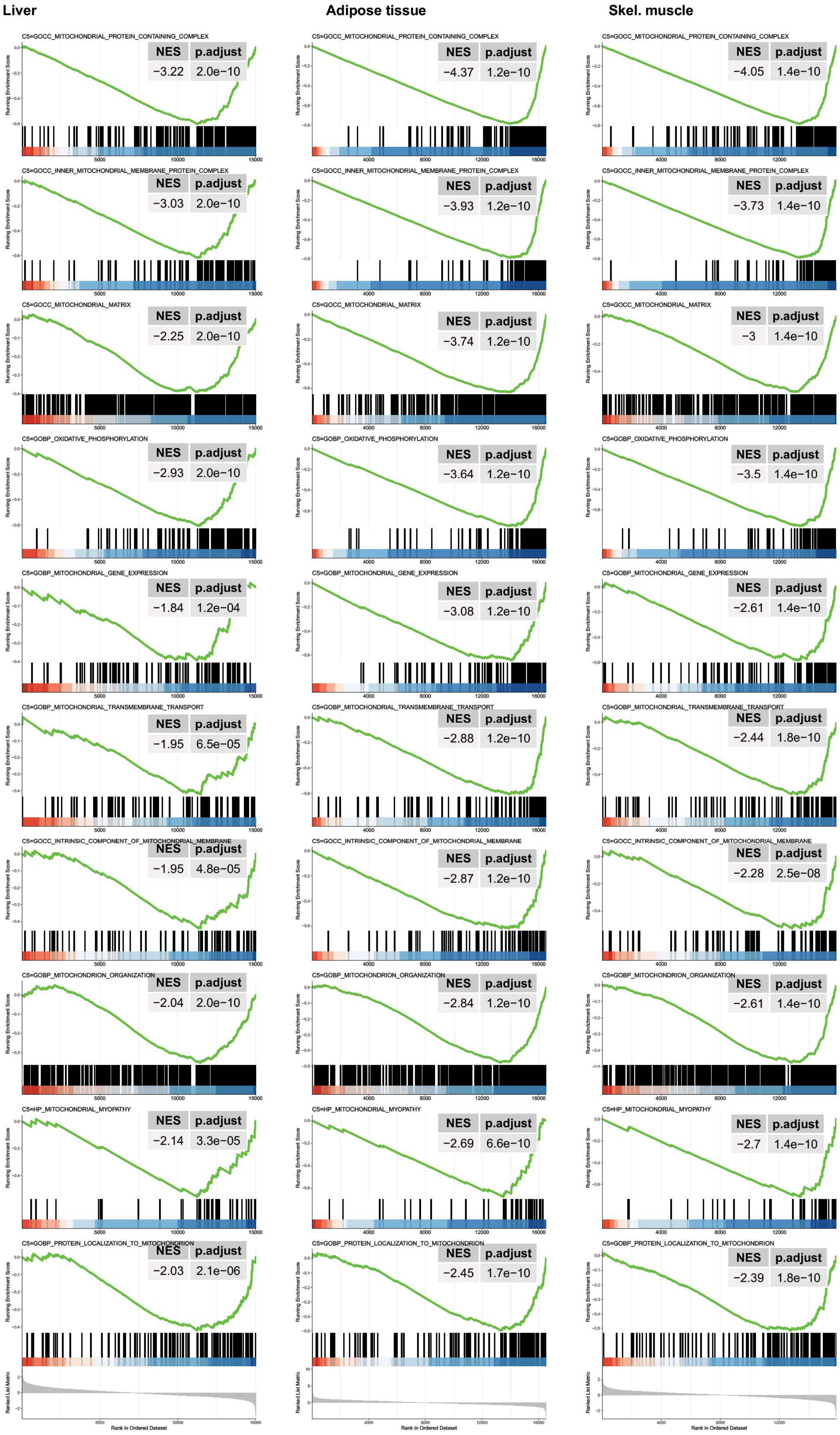
Liver
Adipose tissue
Skel. muscle
C5=GOCC_MITOCHONDRIAL_PROTEIN_CONTAINING_COMPLEX
NES p.adjust
-3.22 2.0e-10
-4.37 1.2e-10
-4.05 1.4e-10
C5=GOCC_INNER_MITOCHONDRIAL_MEMBRANE_PROTEIN_COMPLEX
NES p.adjust
-3.03 2.0e-10
-3.93 1.2e-10
-3.73 1.4e-10
C5=GOCC_MITOCHONDRIAL_MATRIX
NES p.adjust
-2.25 2.0e-10
-3.74 1.2e-10
-3 1.4e-10
C5=GOBP_OXIDATIVE_PHOSPHORYLATION
NES p.adjust
-2.93 2.0e-10
-3.64 1.2e-10
-3.5 1.4e-10
C5=GOBP_MITOCHONDRIAL_GENE_EXPRESSION
NES p.adjust
-1.84 1.2e-04
-3.08 1.2e-10
-2.61 1.4e-10
C5=GOBP_MITOCHONDRIAL_TRANSMEMBRANE_TRANSPORT
NES p.adjust
-1.95 6.5e-05
-2.88 1.2e-10
-2.44 1.8e-10
C5=GOCC_INTRINSIC_COMPONENT_OF_MITOCHONDRIAL_MEMBRANE
NES p.adjust
-1.95 4.8e-05
-2.87 1.2e-10
-2.28 2.5e-08
C5=GOBP_MITOCHONDRION_ORGANIZATION
NES p.adjust
-2.04 2.0e-10
-2.84 1.2e-10
-2.61 1.4e-10
C5=HP_MITOCHONDRIAL_MYOPATHY
NES p.adjust
-2.14 3.3e-05
-2.69 6.6e-10
-2.7 1.4e-10
C5=GOBP_PROTEIN_LOCALIZATION_TO_MITOCHONDRION
NES p.adjust
-2.03 2.1e-06
-2.45 1.7e-10
-2.39 1.8e-10
Running Enrichment Score
Ranked List Metric
Rank in Ordered Dataset

Figure S4. "Mitochondrial structure & function" GSEA profiles in liver, adipose tissue and skeletal muscle from 5 month old *Mlx*KO mice relative to WT controls

Normalized enrichment scores and q values are indicated in the upper right corner of each profile. Data used to generate the ridge plots shown in Figure 6A are re-graphed and included here along with additional representative profiles. See File S1 for a complete list of all gene sets of significance included in this category.

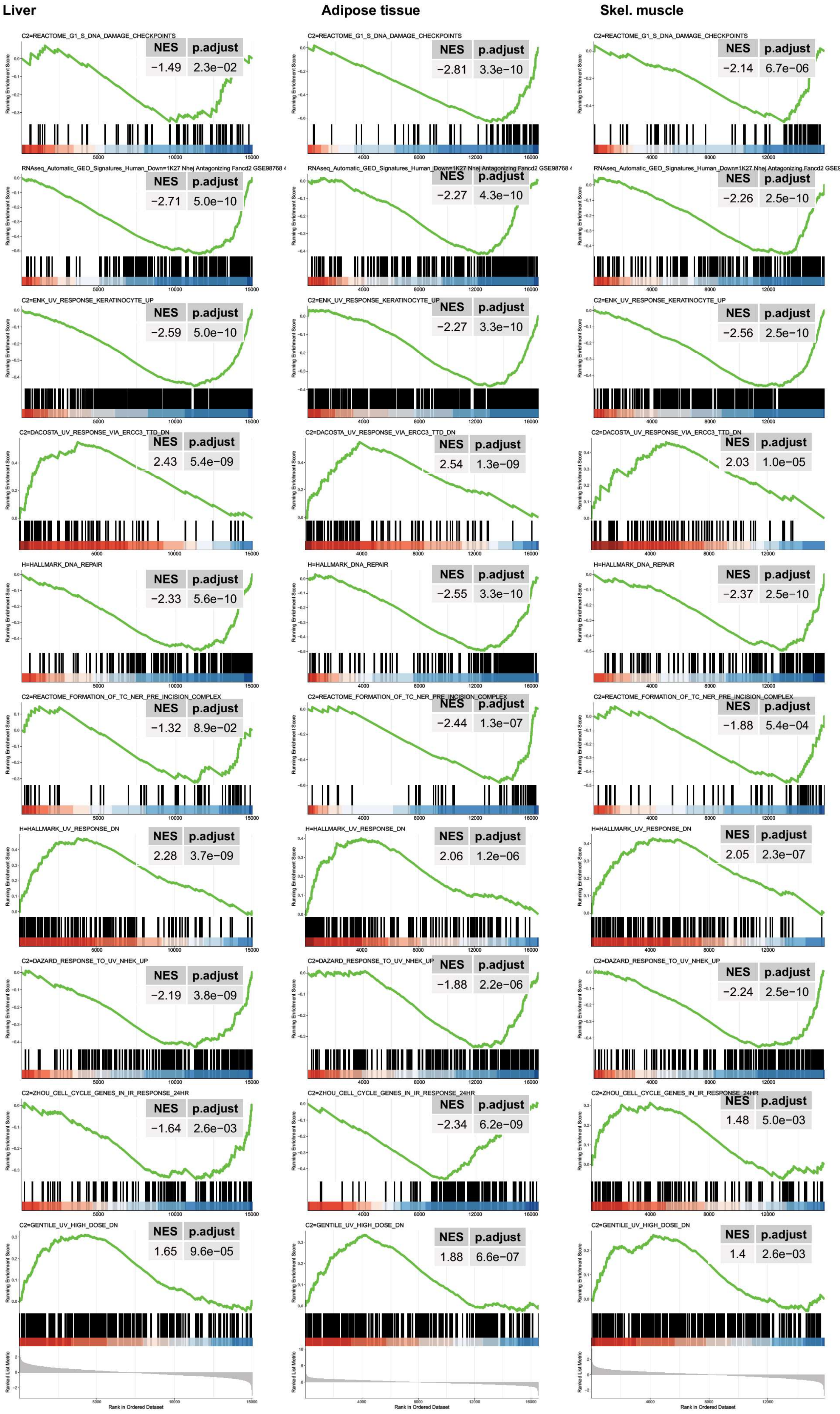
Liver
Adipose tissue
Skel. muscle
C2=REACTOME_G1_S_DNA_DAMAGE_CHECKPOINTS
NES p.adjust
−1.49 2.3e−02
−2.81 3.3e−10
−2.14 6.7e−06
RNAseq_Automatic_GEO_Signatures_Human_Down=1K27 Nhej Antagonizing Fancd2 GSE98768
NES p.adjust
−2.71 5.0e−10
−2.27 4.3e−10
−2.26 2.5e−10
C2=ENK_UV_RESPONSE_KERATINOCYTE_UP
NES p.adjust
−2.59 5.0e−10
−2.27 3.3e−10
−2.56 2.5e−10
C2=DACOSTA_UV_RESPONSE_VIA_ERCC3_TTD_DN
NES p.adjust
2.43 5.4e−09
2.54 1.3e−09
2.03 1.0e−05
H=HALLMARK_DNA_REPAIR
NES p.adjust
−2.33 5.6e−10
−2.55 3.3e−10
−2.37 2.5e−10
C2=REACTOME_FORMATION_OF_TC_NER_PRE_INCISION_COMPLEX
NES p.adjust
−1.32 8.9e−02
−2.44 1.3e−07
−1.88 5.4e−04
H=HALLMARK_UV_RESPONSE_DN
NES p.adjust
2.28 3.7e−09
2.06 1.2e−06
2.05 2.3e−07
C2=DAZARD_RESPONSE_TO_UV_NHEK_UP
NES p.adjust
−2.19 3.8e−09
−1.88 2.2e−06
−2.24 2.5e−10
C2=ZHOU_CELL_CYCLE_GENES_IN_IR_RESPONSE_24HR
NES p.adjust
−1.64 2.6e−03
−2.34 6.2e−09
1.48 5.0e−03
C2=GENTILE_UV_HIGH_DOSE_DN
NES p.adjust
1.65 9.6e−05
1.88 6.6e−07
1.4 2.6e−03
Running Enrichment Score
Ranked List Metric
Rank in Ordered Dataset

Figure S5. "DNA damage response and repair" GSEA profiles in liver, adipose tissue and skeletal muscle from 5 month old *Mlx*KO mice relative to WT controls

Normalized enrichment scores and q values are indicated in the upper right corner of each profile. Data used to generate the ridge plots shown in Figure 6A are re-graphed and included here along with additional representative profiles. See File S1 for a complete list of all gene sets of significance included in this category.

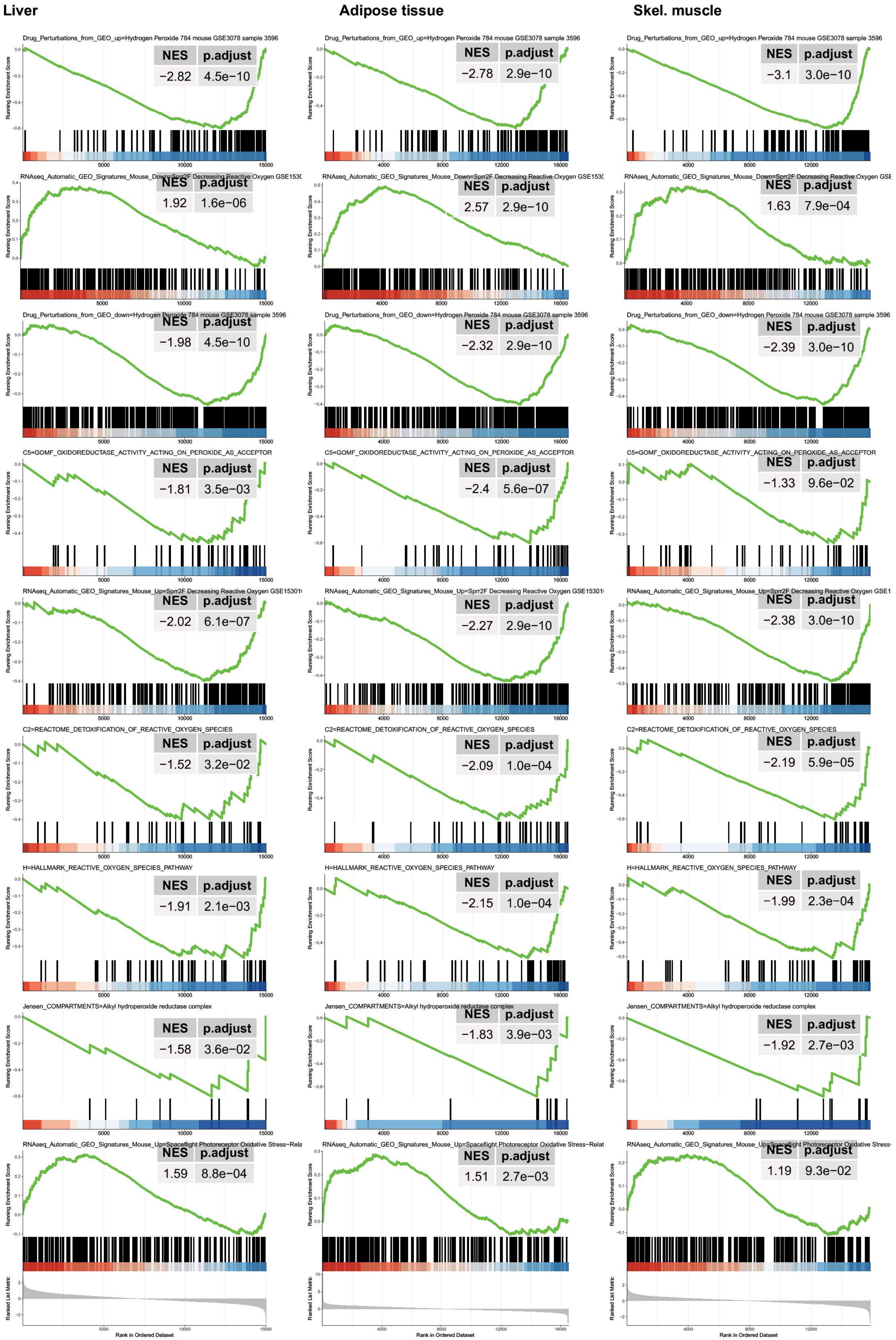

Liver
Adipose tissue
Skel. muscle
Drug_Perturbations_from_GEO_up=Hydrogen Peroxide 784 mouse GSE3078 sample 3596
NES p.adjust
−2.82 4.5e−10
−2.78 2.9e−10
−3.1 3.0e−10
RNAseq_Automatic_GEO_Signatures_Mouse_Down=Sprr2F Decreasing Reactive Oxygen GSE1530
NES p.adjust
1.92 1.6e−06
2.57 2.9e−10
1.63 7.9e−04
Drug_Perturbations_from_GEO_down=Hydrogen Peroxide 784 mouse GSE3078 sample 3596
NES p.adjust
−1.98 4.5e−10
−2.32 2.9e−10
−2.39 3.0e−10
C5=GOMF_OXIDOREDUCTASE_ACTIVITY_ACTING_ON_PEROXIDE_AS_ACCEPTOR
NES p.adjust
−1.81 3.5e−03
−2.4 5.6e−07
−1.33 9.6e−02
RNAseq_Automatic_GEO_Signatures_Mouse_Up=Sprr2F Decreasing Reactive Oxygen GSE15301
NES p.adjust
−2.02 6.1e−07
−2.27 2.9e−10
−2.38 3.0e−10
C2=REACTOME_DETOXIFICATION_OF_REACTIVE_OXYGEN_SPECIES
NES p.adjust
−1.52 3.2e−02
−2.09 1.0e−04
−2.19 5.9e−05
H=HALLMARK_REACTIVE_OXYGEN_SPECIES_PATHWAY
NES p.adjust
−1.91 2.1e−03
−2.15 1.0e−04
−1.99 2.3e−04
Jensen_COMPARTMENTS=Alkyl hydroperoxide reductase complex
NES p.adjust
−1.58 3.6e−02
−1.83 3.9e−03
−1.92 2.7e−03
RNAseq_Automatic_GEO_Signatures_Mouse_Up=Spaceflight Photoreceptor Oxidative Stress−Rela
NES p.adjust
1.59 8.8e−04
1.51 2.7e−03
1.19 9.3e−02
Running Enrichment Score
Ranked List Metric
Rank in Ordered Dataset

Figure S6. “Oxidative stress response” GSEA profiles in liver, adipose tissue and skeletal muscle from 5 month old *Mlx*KO mice relative to WT controls

Normalized enrichment scores and q values are indicated in the upper right corner of each profile. Data used to generate the ridge plots shown in Figure 6A are re-graphed and included here along with additional representative profiles. See File S1 for a complete list of all gene sets of significance included in this category.

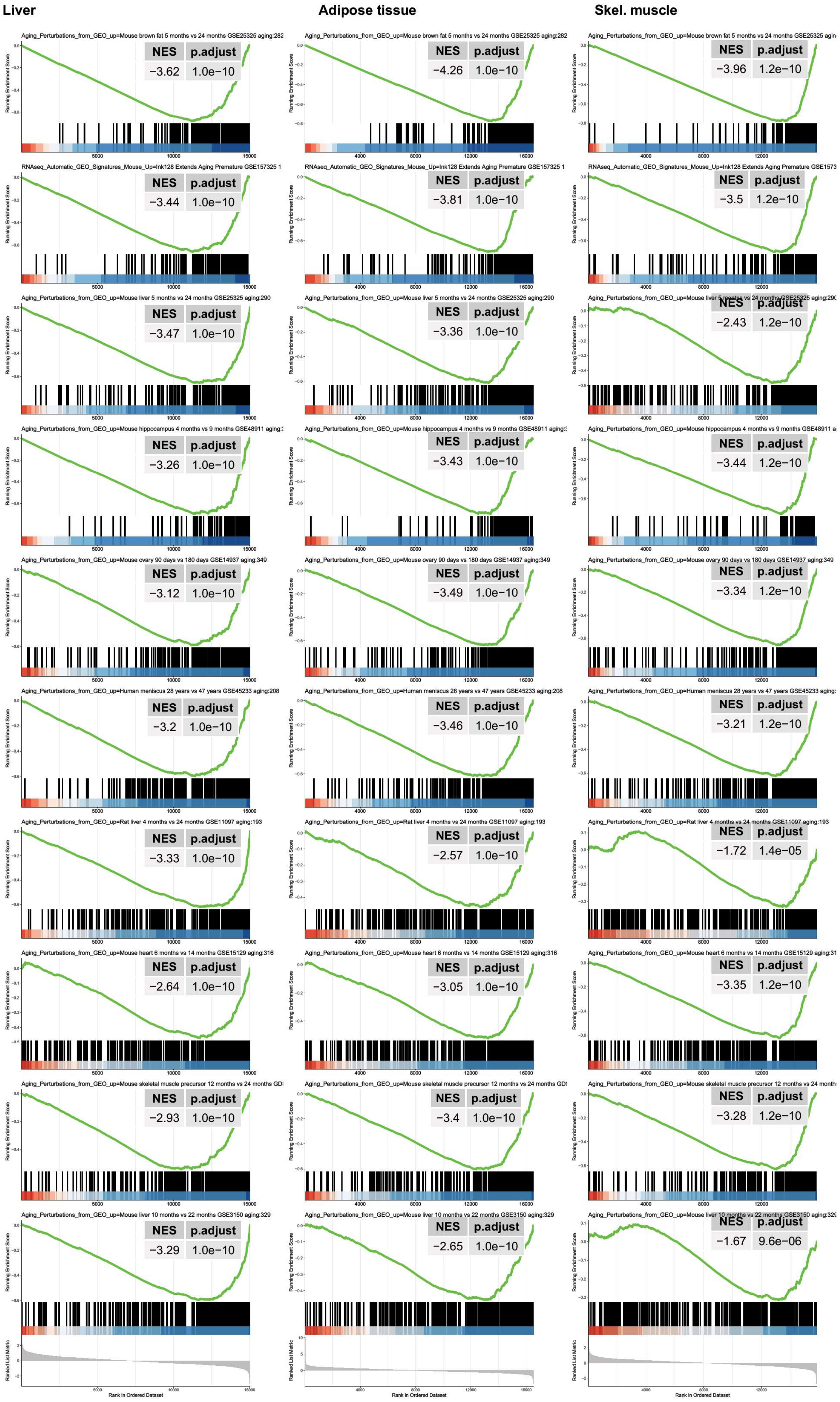
Liver
Adipose tissue
Skel. muscle
Aging_Perturbations_from_GEO_up=Mouse brown fat 5 months vs 24 months GSE25325 aging:282
NES p.adjust
−3.62 1.0e−10
−4.26 1.0e−10
−3.96 1.2e−10
RNAseq_Automatic_GEO_Signatures_Mouse_Up=Ink128 Extends Aging Premature GSE157325 1
NES p.adjust
−3.44 1.0e−10
−3.81 1.0e−10
−3.5 1.2e−10
Aging_Perturbations_from_GEO_up=Mouse liver 5 months vs 24 months GSE25325 aging:290
NES p.adjust
−3.47 1.0e−10
−3.36 1.0e−10
−2.43 1.2e−10
Aging_Perturbations_from_GEO_up=Mouse hippocampus 4 months vs 9 months GSE48911 aging:
NES p.adjust
−3.26 1.0e−10
−3.43 1.0e−10
−3.44 1.2e−10
Aging_Perturbations_from_GEO_up=Mouse ovary 90 days vs 180 days GSE14937 aging:349
NES p.adjust
−3.12 1.0e−10
−3.49 1.0e−10
−3.34 1.2e−10
Aging_Perturbations_from_GEO_up=Human meniscus 28 years vs 47 years GSE45233 aging:208
NES p.adjust
−3.2 1.0e−10
−3.46 1.0e−10
−3.21 1.2e−10
Aging_Perturbations_from_GEO_up=Rat liver 4 months vs 24 months GSE11097 aging:193
NES p.adjust
−3.33 1.0e−10
−2.57 1.0e−10
−1.72 1.4e−05
Aging_Perturbations_from_GEO_up=Mouse heart 6 months vs 14 months GSE15129 aging:316
NES p.adjust
−2.64 1.0e−10
−3.05 1.0e−10
−3.35 1.2e−10
Aging_Perturbations_from_GEO_up=Mouse skeletal muscle precursor 12 months vs 24 months GD
NES p.adjust
−2.93 1.0e−10
−3.4 1.0e−10
−3.28 1.2e−10
Aging_Perturbations_from_GEO_up=Mouse liver 10 months vs 22 months GSE3150 aging:329
NES p.adjust
−3.29 1.0e−10
−2.65 1.0e−10
−1.67 9.6e−06
Running Enrichment Score
Ranked List Metric
Rank in Ordered Dataset

Figure S7. "Aging" GSEA profiles in liver, adipose tissue and skeletal muscle from 5 month old *Mlx*KO mice relative to WT controls

Normalized enrichment scores and q values are indicated in the upper right corner of each profile. Data used to generate the ridge plots shown in Figure 6A are re-graphed and included here along with additional representative profiles. See File S1 for a complete list of all gene sets of significance included in this category.

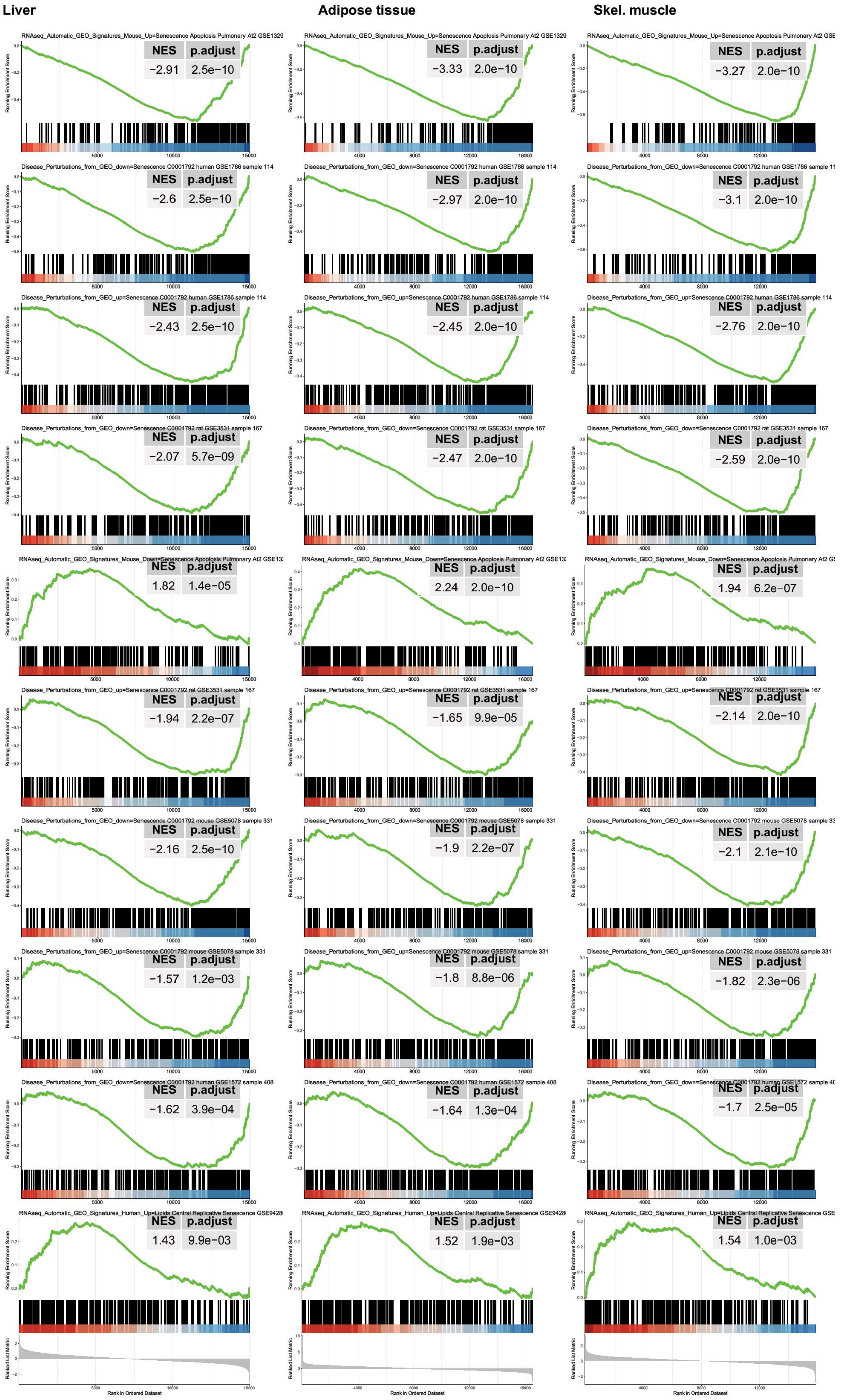

Liver
Adipose tissue
Skel. muscle
RNAseq_Automatic_GEO_Signatures_Mouse_Up=Senescence Apoptosis Pulmonary At2 GSE1329
NES p.adjust
−2.91 2.5e−10
−3.33 2.0e−10
−3.27 2.0e−10
Disease_Perturbations_from_GEO_down=Senescence C0001792 human GSE1786 sample 114
NES p.adjust
−2.6 2.5e−10
−2.97 2.0e−10
−3.1 2.0e−10
Disease_Perturbations_from_GEO_up=Senescence C0001792 human GSE1786 sample 114
NES p.adjust
−2.43 2.5e−10
−2.45 2.0e−10
−2.76 2.0e−10
Disease_Perturbations_from_GEO_down=Senescence C0001792 rat GSE3531 sample 167
NES p.adjust
−2.07 5.7e−09
−2.47 2.0e−10
−2.59 2.0e−10
RNAseq_Automatic_GEO_Signatures_Mouse_Down=Senescence Apoptosis Pulmonary At2 GSE13
NES p.adjust
1.82 1.4e−05
2.24 2.0e−10
1.94 6.2e−07
Disease_Perturbations_from_GEO_up=Senescence C0001792 rat GSE3531 sample 167
NES p.adjust
−1.94 2.2e−07
−1.65 9.9e−05
−2.14 2.0e−10
Disease_Perturbations_from_GEO_down=Senescence C0001792 mouse GSE5078 sample 331
NES p.adjust
−2.16 2.5e−10
−1.9 2.2e−07
−2.1 2.1e−10
Disease_Perturbations_from_GEO_up=Senescence C0001792 mouse GSE5078 sample 331
NES p.adjust
−1.57 1.2e−03
−1.8 8.8e−06
−1.82 2.3e−06
Disease_Perturbations_from_GEO_down=Senescence C0001792 human GSE1572 sample 408
NES p.adjust
−1.62 3.9e−04
−1.64 1.3e−04
−1.7 2.5e−05
RNAseq_Automatic_GEO_Signatures_Human_Up=Lipids Central Replicative Senescence GSE9428
NES p.adjust
1.43 9.9e−03
1.52 1.9e−03
1.54 1.0e−03
Running Enrichment Score
Ranked List Metric
Rank in Ordered Dataset

Figure S8. “Senescence” GSEA profiles in liver, adipose tissue and skeletal muscle from 5 month old *Mlx*KO mice relative to WT controls

Normalized enrichment scores and q values are indicated in the upper right corner of each profile. Data used to generate the ridge plots shown in Figure 6A are re-graphed and included here along with additional representative profiles. See File S1 for a complete list of all gene sets of significance included in this category.

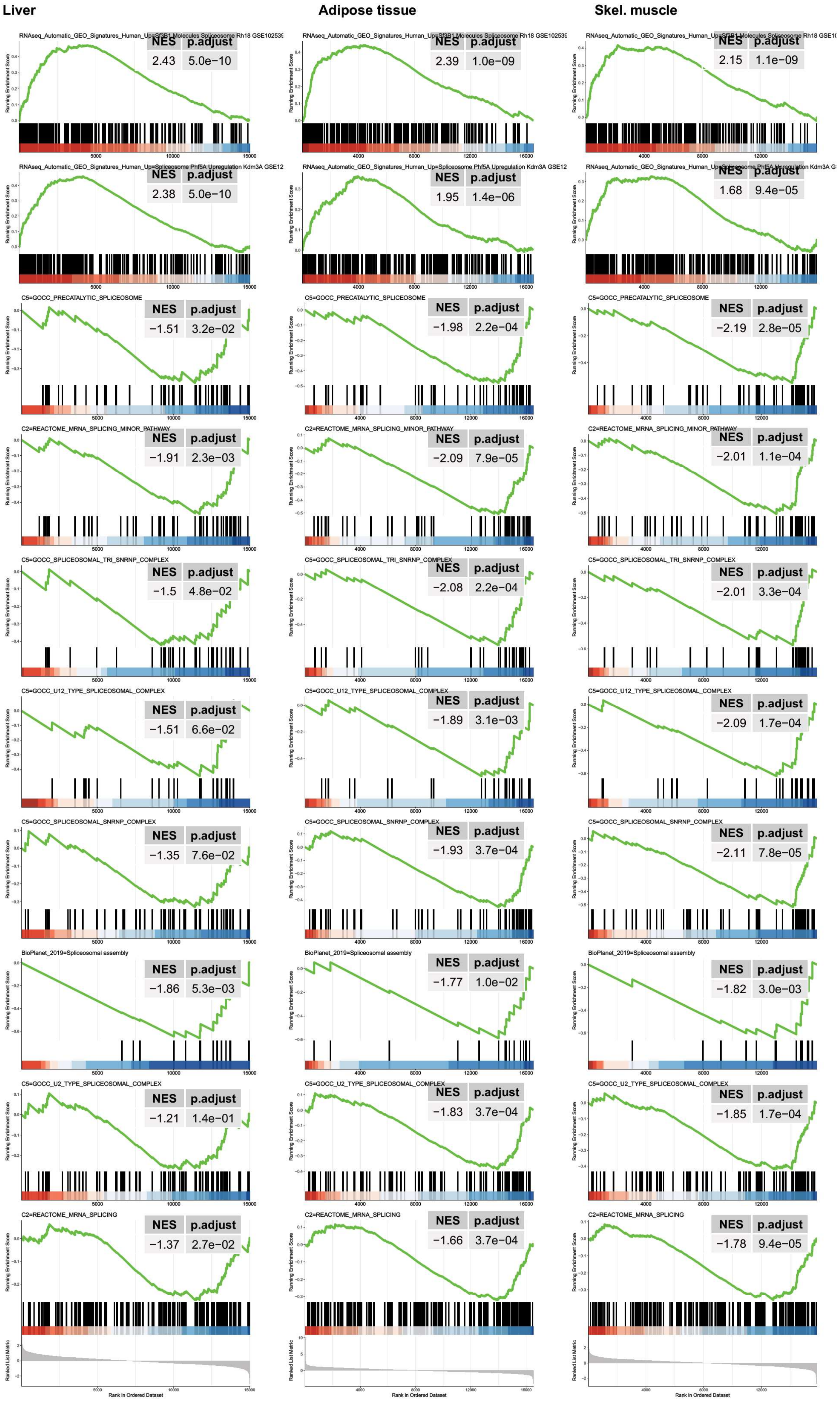
Liver
Adipose tissue
Skel. muscle
RNAseq_Automatic_GEO_Signatures_Human_Up=Sf3B1 Molecules Spliceosome Rh18 GSE102539
NES p.adjust
2.43 5.0e−10
2.39 1.0e−09
2.15 1.1e−09
RNAseq_Automatic_GEO_Signatures_Human_Up=Spliceosome Phf5A Upregulation Kdm3A GSE12
NES p.adjust
2.38 5.0e−10
1.95 1.4e−06
1.68 9.4e−05
C5=GOCC_PRECATALYTIC_SPLICEOSOME
NES p.adjust
−1.51 3.2e−02
−1.98 2.2e−04
−2.19 2.8e−05
C2=REACTOME_MRNA_SPLICING_MINOR_PATHWAY
NES p.adjust
−1.91 2.3e−03
−2.09 7.9e−05
−2.01 1.1e−04
C5=GOCC_SPLICEOSOMAL_TRI_SNRNP_COMPLEX
NES p.adjust
−1.5 4.8e−02
−2.08 2.2e−04
−2.01 3.3e−04
C5=GOCC_U12_TYPE_SPLICEOSOMAL_COMPLEX
NES p.adjust
−1.51 6.6e−02
−1.89 3.1e−03
−2.09 1.7e−04
C5=GOCC_SPLICEOSOMAL_SNRNP_COMPLEX
NES p.adjust
−1.35 7.6e−02
−1.93 3.7e−04
−2.11 7.8e−05
BioPlanet_2019=Spliceosomal assembly
NES p.adjust
−1.86 5.3e−03
−1.77 1.0e−02
−1.82 3.0e−03
C5=GOCC_U2_TYPE_SPLICEOSOMAL_COMPLEX
NES p.adjust
−1.21 1.4e−01
−1.83 3.7e−04
−1.85 1.7e−04
C2=REACTOME_MRNA_SPLICING
NES p.adjust
−1.37 2.7e−02
−1.66 3.7e−04
−1.78 9.4e−05
Running Enrichment Score
Ranked List Metric
Rank in Ordered Dataset

Figure S9. "mRNA splicing" GSEA profiles in liver, adipose tissue and skeletal muscle from 5 month old *Mlx*KO mice relative to WT controls

Normalized enrichment scores and q values are indicated in the upper right corner of each profile. Data used to generate the ridge plots shown in Figure 6A are re-graphed and included here along with additional representative profiles. See File S1 for a complete list of all gene sets of significance included in this category.

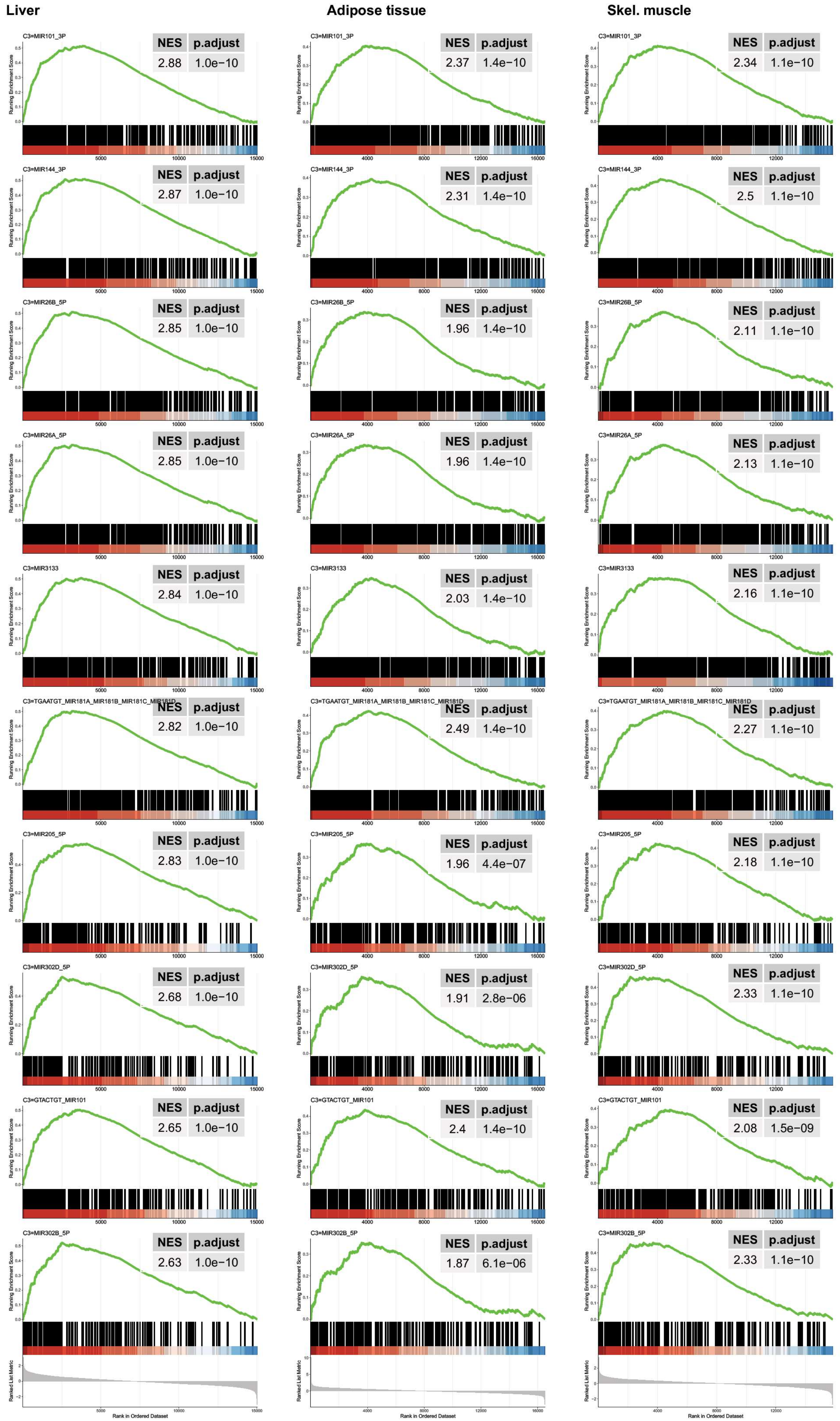
Liver
Adipose tissue
Skel. muscle
C3=MIR101_3P
NES p.adjust
2.88 1.0e−10
2.37 1.4e−10
2.34 1.1e−10
C3=MIR144_3P
2.87 1.0e−10
2.31 1.4e−10
2.5 1.1e−10
C3=MIR26B_5P
2.85 1.0e−10
1.96 1.4e−10
2.11 1.1e−10
C3=MIR26A_5P
2.85 1.0e−10
1.96 1.4e−10
2.13 1.1e−10
C3=MIR3133
2.84 1.0e−10
2.03 1.4e−10
2.16 1.1e−10
C3=TGAATGT_MIR181A_MIR181B_MIR181C_MIR181D
2.82 1.0e−10
2.49 1.4e−10
2.27 1.1e−10
C3=MIR205_5P
2.83 1.0e−10
1.96 4.4e−07
2.18 1.1e−10
C3=MIR302D_5P
2.68 1.0e−10
1.91 2.8e−06
2.33 1.1e−10
C3=GTACTGT_MIR101
2.65 1.0e−10
2.4 1.4e−10
2.08 1.5e−09
C3=MIR302B_5P
2.63 1.0e−10
1.87 6.1e−06
2.33 1.1e−10
Running Enrichment Score
Ranked List Metric
Rank in Ordered Dataset

Figure S10. "miRNA Targets" GSEA profiles in liver, adipose tissue and skeletal muscle from 5 month old *Mlx*KO mice relative to WT controls.
Normalized enrichment scores (NESs) and q values are indicated in the upper right corner of each profile. Data used to generate the ridgeline plots shown in Figure 6B are re-graphed and included here along with additional representative GSEA profiles. See File S2 for a complete list of all gene sets of significance included in this category.

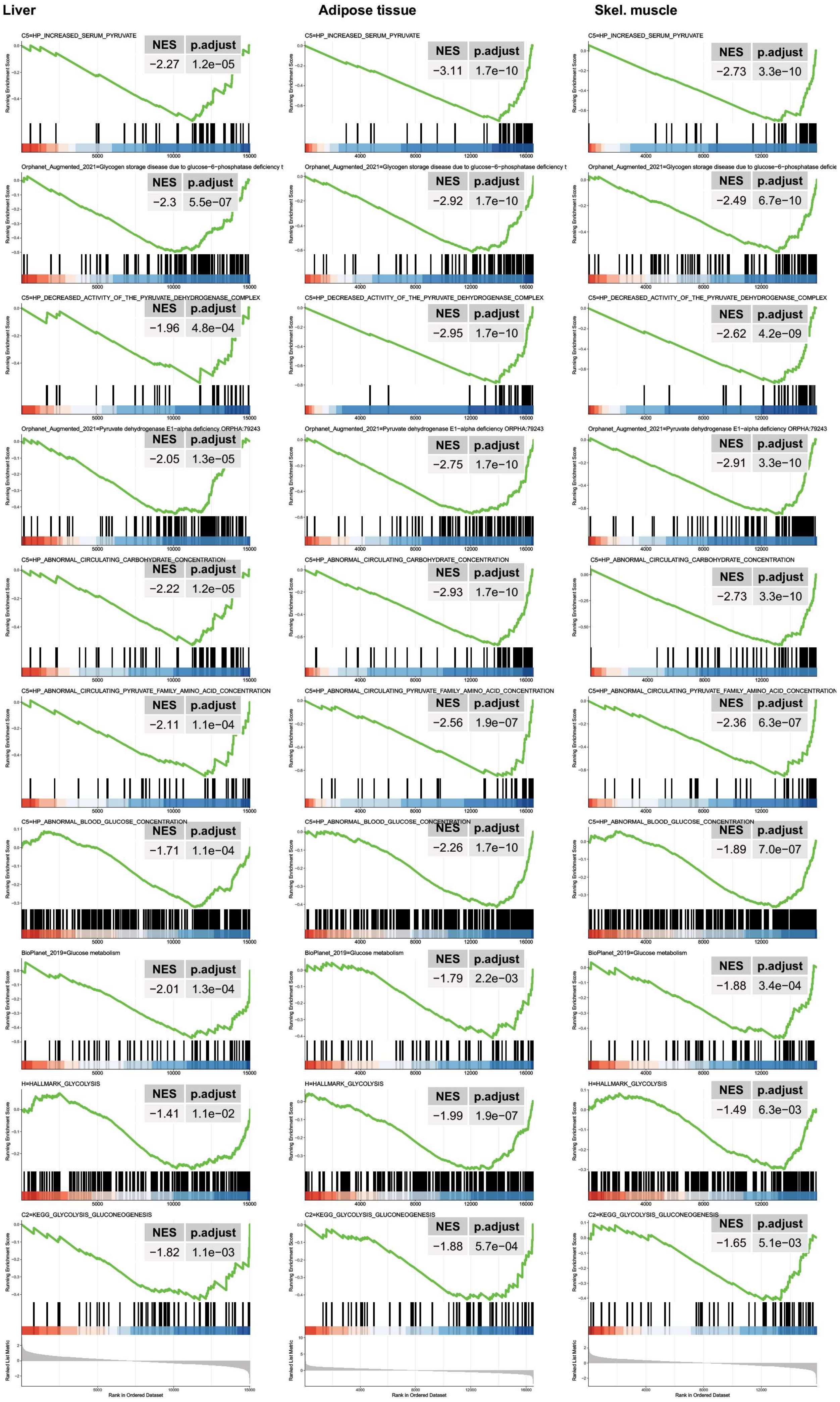

Liver
Adipose tissue
Skel. muscle
C5=HP_INCREASED_SERUM_PYRUVATE
NES −2.27 p.adjust 1.2e−05
NES −3.11 p.adjust 1.7e−10
NES −2.73 p.adjust 3.3e−10
Orphanet_Augmented_2021=Glycogen storage disease due to glucose−6−phosphatase deficiency t
NES −2.3 p.adjust 5.5e−07
NES −2.92 p.adjust 1.7e−10
NES −2.49 p.adjust 6.7e−10
C5=HP_DECREASED_ACTIVITY_OF_THE_PYRUVATE_DEHYDROGENASE_COMPLEX
NES −1.96 p.adjust 4.8e−04
NES −2.95 p.adjust 1.7e−10
NES −2.62 p.adjust 4.2e−09
Orphanet_Augmented_2021=Pyruvate dehydrogenase E1−alpha deficiency ORPHA:79243
NES −2.05 p.adjust 1.3e−05
NES −2.75 p.adjust 1.7e−10
NES −2.91 p.adjust 3.3e−10
C5=HP_ABNORMAL_CIRCULATING_CARBOHYDRATE_CONCENTRATION
NES −2.22 p.adjust 1.2e−05
NES −2.93 p.adjust 1.7e−10
NES −2.73 p.adjust 3.3e−10
C5=HP_ABNORMAL_CIRCULATING_PYRUVATE_FAMILY_AMINO_ACID_CONCENTRATION
NES −2.11 p.adjust 1.1e−04
NES −2.56 p.adjust 1.9e−07
NES −2.36 p.adjust 6.3e−07
C5=HP_ABNORMAL_BLOOD_GLUCOSE_CONCENTRATION
NES −1.71 p.adjust 1.1e−04
NES −2.26 p.adjust 1.7e−10
NES −1.89 p.adjust 7.0e−07
BioPlanet_2019=Glucose metabolism
NES −2.01 p.adjust 1.3e−04
NES −1.79 p.adjust 2.2e−03
NES −1.88 p.adjust 3.4e−04
H=HALLMARK_GLYCOLYSIS
NES −1.41 p.adjust 1.1e−02
NES −1.99 p.adjust 1.9e−07
NES −1.49 p.adjust 6.3e−03
C2=KEGG_GLYCOLYSIS_GLUCONEOGENESIS
NES −1.82 p.adjust 1.1e−03
NES −1.88 p.adjust 5.7e−04
NES −1.65 p.adjust 5.1e−03
Running Enrichment Score
Ranked List Metric
Rank in Ordered Dataset

Figure S11. "Glycolysis-related" GSEA profiles in liver, adipose tissue and skeletal muscle from 5 month old *Mlx*KO mice relative to WT controls.

Normalized enrichment scores (NESs) and q values are indicated in the upper right corner of each profile. Data used to generate the ridgeline plots shown in Figure 6B are re-graphed and included here along with additional representative GSEA profiles. See File S2 for a complete list of all gene sets of significance included in this category.

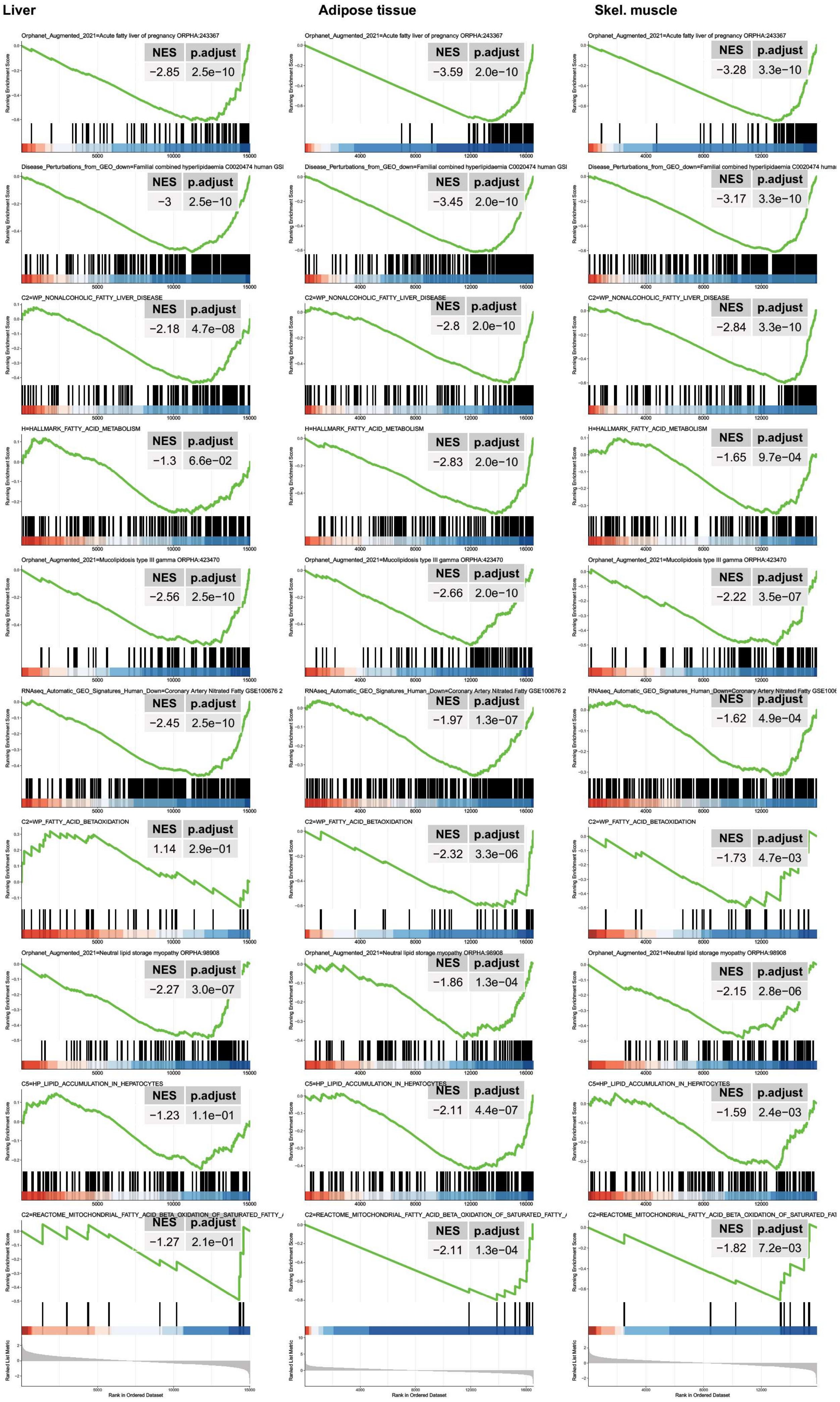
Liver
Adipose tissue
Skel. muscle
Orphanet_Augmented_2021=Acute fatty liver of pregnancy ORPHA:243367
NES p.adjust
−2.85 2.5e−10
−3.59 2.0e−10
−3.28 3.3e−10
Disease_Perturbations_from_GEO_down=Familial combined hyperlipidaemia C0020474 human GSI
NES p.adjust
−3 2.5e−10
−3.45 2.0e−10
−3.17 3.3e−10
C2=WP_NONALCOHOLIC_FATTY_LIVER_DISEASE
NES p.adjust
−2.18 4.7e−08
−2.8 2.0e−10
−2.84 3.3e−10
H=HALLMARK_FATTY_ACID_METABOLISM
NES p.adjust
−1.3 6.6e−02
−2.83 2.0e−10
−1.65 9.7e−04
Orphanet_Augmented_2021=Mucolipidosis type III gamma ORPHA:423470
NES p.adjust
−2.56 2.5e−10
−2.66 2.0e−10
−2.22 3.5e−07
RNAseq_Automatic_GEO_Signatures_Human_Down=Coronary Artery Nitrated Fatty GSE100676 2
NES p.adjust
−2.45 2.5e−10
−1.97 1.3e−07
−1.62 4.9e−04
C2=WP_FATTY_ACID_BETAOXIDATION
NES p.adjust
1.14 2.9e−01
−2.32 3.3e−06
−1.73 4.7e−03
Orphanet_Augmented_2021=Neutral lipid storage myopathy ORPHA:98908
NES p.adjust
−2.27 3.0e−07
−1.86 1.3e−04
−2.15 2.8e−06
C5=HP_LIPID_ACCUMULATION_IN_HEPATOCYTES
NES p.adjust
−1.23 1.1e−01
−2.11 4.4e−07
−1.59 2.4e−03
C2=REACTOME_MITOCHONDRIAL_FATTY_ACID_BETA_OXIDATION_OF_SATURATED_FATTY_
NES p.adjust
−1.27 2.1e−01
−2.11 1.3e−04
−1.82 7.2e−03
Running Enrichment Score
Ranked List Metric
Rank in Ordered Dataset

Figure S12. "Lipid metabolism" GSEA profiles in liver, adipose tissue and skeletal muscle from 5 month old *Mlx*KO mice relative to WT controls.
Normalized enrichment scores (NESs) and q values are indicated in the upper right corner of each profile. Data used to generate the ridgeline plots shown in Figure 6B are re-graphed and included here along with additional representative GSEA profiles. See File S2 for a complete list of all gene sets of significance included in this category.

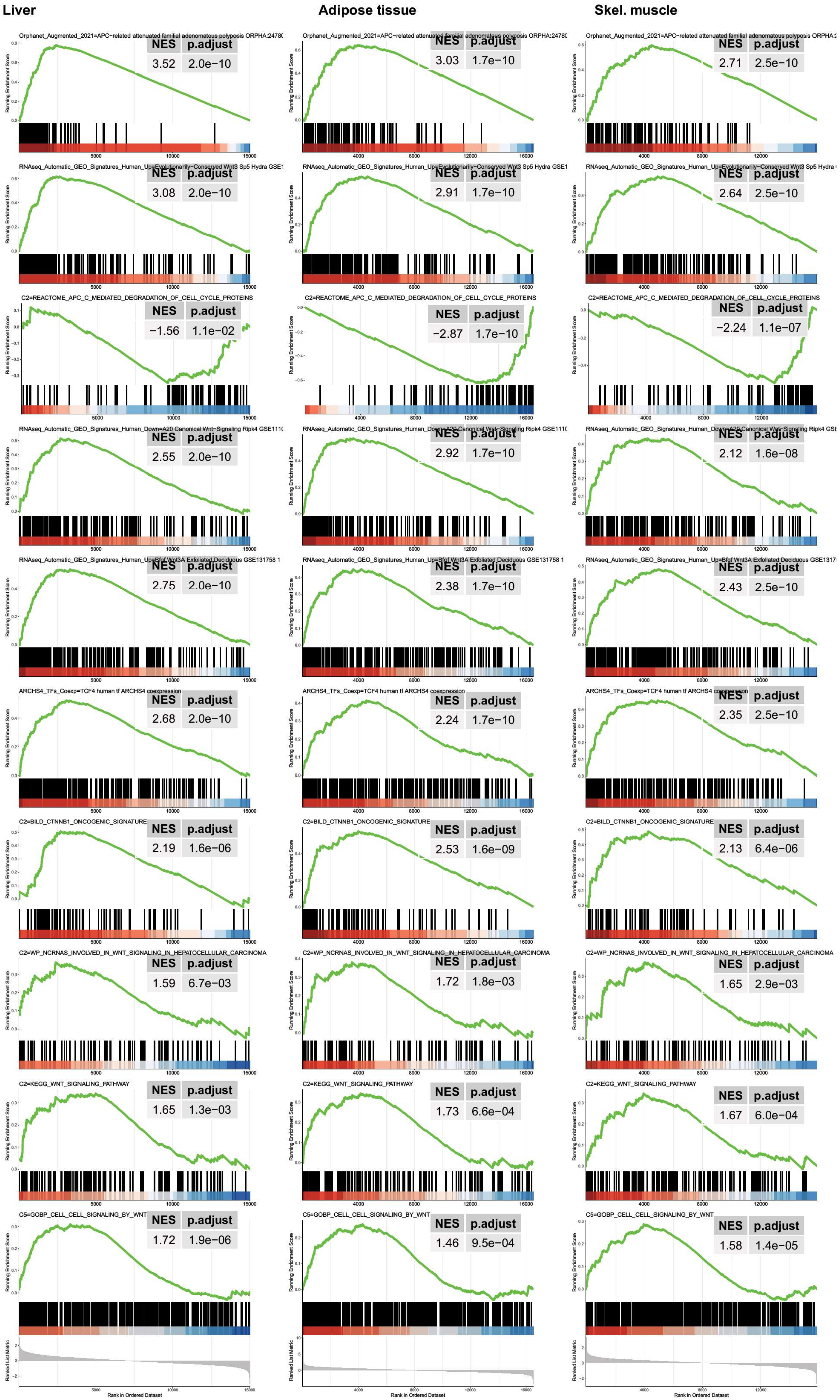
Liver
Adipose tissue
Skel. muscle
Orphanet_Augmented_2021=APC−related attenuated familial adenomatous polyposis ORPHA:24780
NES p.adjust
3.52 2.0e−10
3.03 1.7e−10
2.71 2.5e−10
RNAseq_Automatic_GEO_Signatures_Human_Up=Evolutionarily−Conserved Wnt3 Sp5 Hydra GSE1
3.08 2.0e−10
2.91 1.7e−10
2.64 2.5e−10
C2=REACTOME_APC_C_MEDIATED_DEGRADATION_OF_CELL_CYCLE_PROTEINS
−1.56 1.1e−02
−2.87 1.7e−10
−2.24 1.1e−07
RNAseq_Automatic_GEO_Signatures_Human_Down=A20 Canonical Wnt−Signaling Ripk4 GSE1110
2.55 2.0e−10
2.92 1.7e−10
2.12 1.6e−08
RNAseq_Automatic_GEO_Signatures_Human_Up=Bfgf Wnt3A Exfoliated Deciduous GSE131758 1
2.75 2.0e−10
2.38 1.7e−10
2.43 2.5e−10
ARCHS4_TFs_Coexp=TCF4 human tf ARCHS4 coexpression
2.68 2.0e−10
2.24 1.7e−10
2.35 2.5e−10
C2=BILD_CTNNB1_ONCOGENIC_SIGNATURE
2.19 1.6e−06
2.53 1.6e−09
2.13 6.4e−06
C2=WP_NCRNAS_INVOLVED_IN_WNT_SIGNALING_IN_HEPATOCELLULAR_CARCINOMA
1.59 6.7e−03
1.72 1.8e−03
1.65 2.9e−03
C2=KEGG_WNT_SIGNALING_PATHWAY
1.65 1.3e−03
1.73 6.6e−04
1.67 6.0e−04
C5=GOBP_CELL_CELL_SIGNALING_BY_WNT
1.72 1.9e−06
1.46 9.5e−04
1.58 1.4e−05
Running Enrichment Score
Ranked List Metric
Rank in Ordered Dataset

Figure S13. "Wnt/β-catenin/Tcf signaling" GSEA profiles in liver, adipose tissue and skeletal muscle from 5 month old *Mlx*KO mice relative to WT controls.

Normalized enrichment scores (NESs) and q values are indicated in the upper right corner of each profile. Data used to generate the ridgeline plots shown in Figure 6B are re-graphed and included here along with additional representative GSEA profiles. See File S2 for a complete list of all gene sets of significance included in this category.

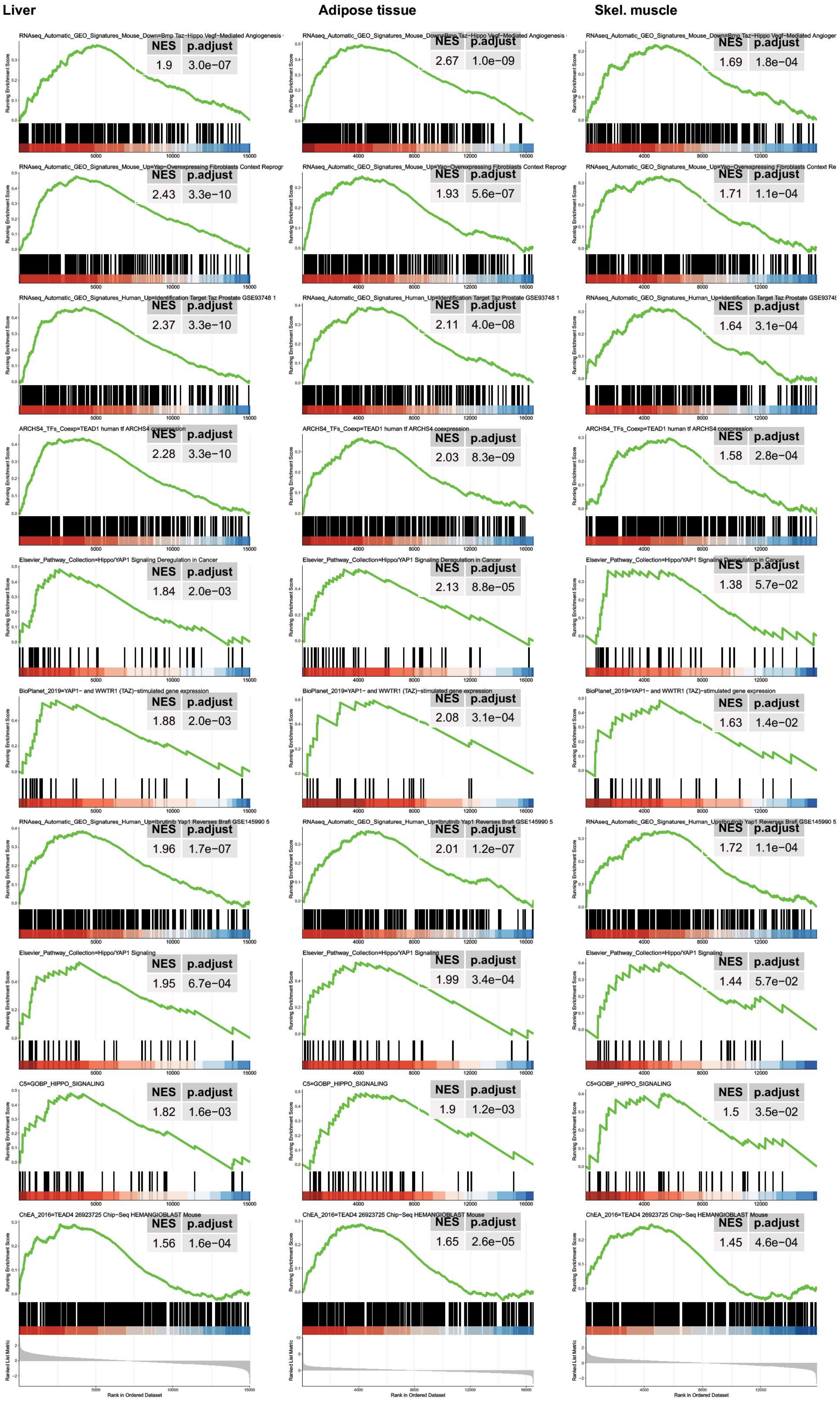

Liver
Adipose tissue
Skel. muscle
RNAseq_Automatic_GEO_Signatures_Mouse_Down=Bmp Taz−Hippo Vegf−Mediated Angiogenesis
NES p.adjust
1.9 3.0e−07
2.67 1.0e−09
1.69 1.8e−04
RNAseq_Automatic_GEO_Signatures_Mouse_Up=Yap−Overexpressing Fibroblasts Context Reprogr
2.43 3.3e−10
1.93 5.6e−07
1.71 1.1e−04
RNAseq_Automatic_GEO_Signatures_Human_Up=Identification Target Taz Prostate GSE93748 1
2.37 3.3e−10
2.11 4.0e−08
1.64 3.1e−04
ARCHS4_TFs_Coexp=TEAD1 human tf ARCHS4 coexpression
2.28 3.3e−10
2.03 8.3e−09
1.58 2.8e−04
Elsevier_Pathway_Collection=Hippo/YAP1 Signaling Deregulation in Cancer
1.84 2.0e−03
2.13 8.8e−05
1.38 5.7e−02
BioPlanet_2019=YAP1− and WWTR1 (TAZ)−stimulated gene expression
1.88 2.0e−03
2.08 3.1e−04
1.63 1.4e−02
RNAseq_Automatic_GEO_Signatures_Human_Up=Ibrutinib Yap1 Reverses Brafi GSE145990 5
1.96 1.7e−07
2.01 1.2e−07
1.72 1.1e−04
Elsevier_Pathway_Collection=Hippo/YAP1 Signaling
1.95 6.7e−04
1.99 3.4e−04
1.44 5.7e−02
C5=GOBP_HIPPO_SIGNALING
1.82 1.6e−03
1.9 1.2e−03
1.5 3.5e−02
ChEA_2016=TEAD4 26923725 Chip−Seq HEMANGIOBLAST Mouse
1.56 1.6e−04
1.65 2.6e−05
1.45 4.6e−04
Running Enrichment Score
Ranked List Metric
Rank in Ordered Dataset

Figure S14. “Hippo/YAP/TAZ signaling” GSEA profiles in liver, adipose tissue and skeletal muscle from 5 month old *Mlx*KO mice relative to WT controls.
Normalized enrichment scores (NESs) and q values are indicated in the upper right corner of each profile. Data used to generate the ridgeline plots shown in Figure 6B are re-graphed and included here along with additional representative GSEA profiles. See File S2 for a complete list of all gene sets of significance included in this category.

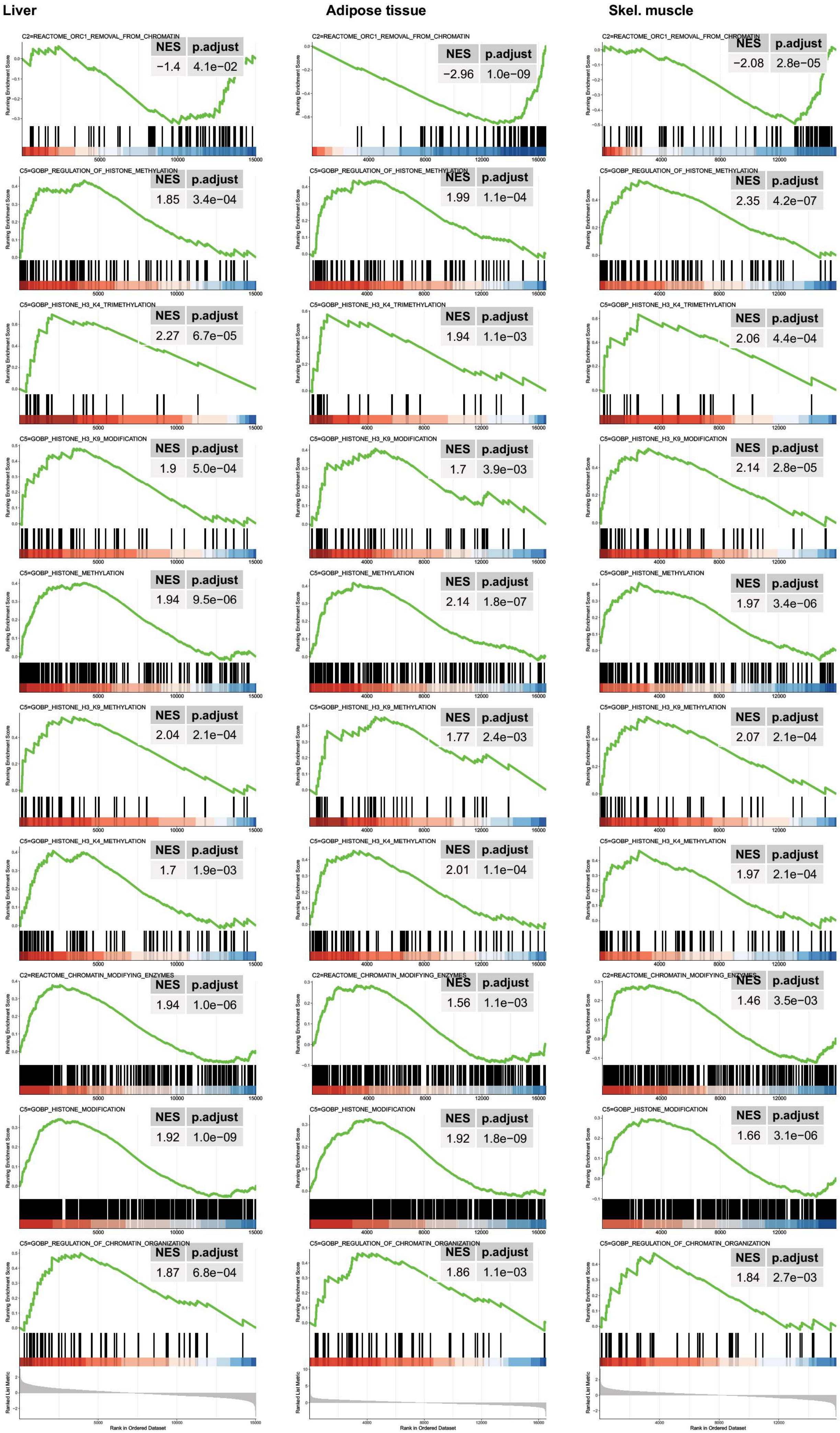

Liver
Adipose tissue
Skel. muscle
C2=REACTOME_ORC1_REMOVAL_FROM_CHROMATIN
NES p.adjust
−1.4 4.1e−02
−2.96 1.0e−09
−2.08 2.8e−05
C5=GOBP_REGULATION_OF_HISTONE_METHYLATION
1.85 3.4e−04
1.99 1.1e−04
2.35 4.2e−07
C5=GOBP_HISTONE_H3_K4_TRIMETHYLATION
2.27 6.7e−05
1.94 1.1e−03
2.06 4.4e−04
C5=GOBP_HISTONE_H3_K9_MODIFICATION
1.9 5.0e−04
1.7 3.9e−03
2.14 2.8e−05
C5=GOBP_HISTONE_METHYLATION
1.94 9.5e−06
2.14 1.8e−07
1.97 3.4e−06
C5=GOBP_HISTONE_H3_K9_METHYLATION
2.04 2.1e−04
1.77 2.4e−03
2.07 2.1e−04
C5=GOBP_HISTONE_H3_K4_METHYLATION
1.7 1.9e−03
2.01 1.1e−04
1.97 2.1e−04
C2=REACTOME_CHROMATIN_MODIFYING_ENZYMES
1.94 1.0e−06
1.56 1.1e−03
1.46 3.5e−03
C5=GOBP_HISTONE_MODIFICATION
1.92 1.0e−09
1.92 1.8e−09
1.66 3.1e−06
C5=GOBP_REGULATION_OF_CHROMATIN_ORGANIZATION
1.87 6.8e−04
1.86 1.1e−03
1.84 2.7e−03
Running Enrichment Score
Ranked List Metric
Rank in Ordered Dataset

Figure S15. “Chromatin/histone modification” GSEA profiles in liver, adipose tissue and skeletal muscle from 5 month old *Mlx*KO mice relative to WT controls.

Normalized enrichment scores (NESs) and q values are indicated in the upper right corner of each profile. Data used to generate the ridgeline plots shown in Figure 6B are re-graphed and included here along with additional representative GSEA profiles. See File S2 for a complete list of all gene sets of significance included in this category.

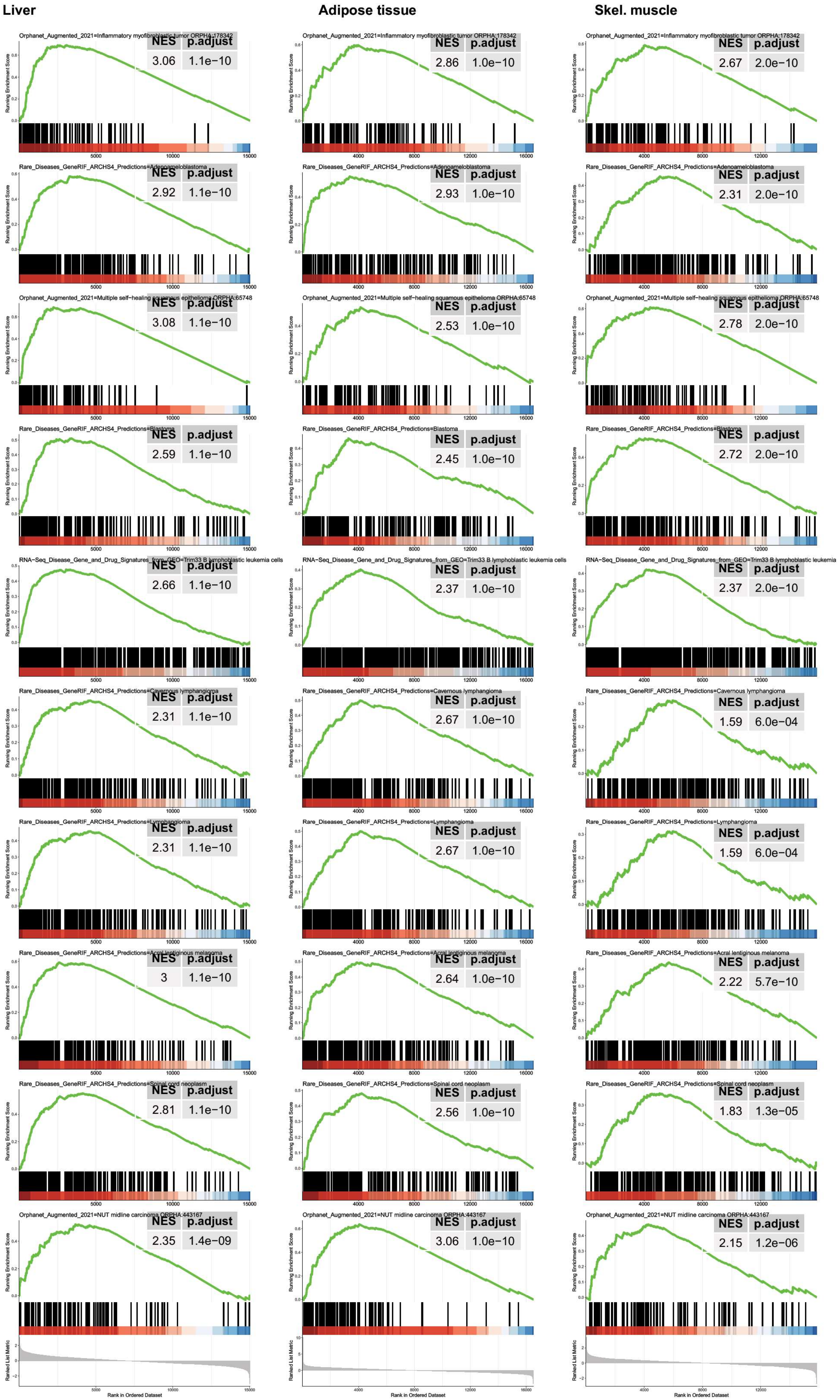
Liver
Adipose tissue
Skel. muscle
Orphanet_Augmented_2021=Inflammatory myofibroblastic tumor ORPHA:178342
NES p.adjust
3.06 1.1e−10
2.86 1.0e−10
2.67 2.0e−10
Rare_Diseases_GeneRIF_ARCHS4_Predictions=Adenoameloblastoma
2.92 1.1e−10
2.93 1.0e−10
2.31 2.0e−10
Orphanet_Augmented_2021=Multiple self−healing squamous epithelioma ORPHA:65748
3.08 1.1e−10
2.53 1.0e−10
2.78 2.0e−10
Rare_Diseases_GeneRIF_ARCHS4_Predictions=Blastoma
2.59 1.1e−10
2.45 1.0e−10
2.72 2.0e−10
RNA−Seq_Disease_Gene_and_Drug_Signatures_from_GEO=Trim33 B lymphoblastic leukemia cells
2.66 1.1e−10
2.37 1.0e−10
2.37 2.0e−10
Rare_Diseases_GeneRIF_ARCHS4_Predictions=Cavernous lymphangioma
2.31 1.1e−10
2.67 1.0e−10
1.59 6.0e−04
Rare_Diseases_GeneRIF_ARCHS4_Predictions=Lymphangioma
2.31 1.1e−10
2.67 1.0e−10
1.59 6.0e−04
Rare_Diseases_GeneRIF_ARCHS4_Predictions=Acral lentiginous melanoma
3 1.1e−10
2.64 1.0e−10
2.22 5.7e−10
Rare_Diseases_GeneRIF_ARCHS4_Predictions=Spinal cord neoplasm
2.81 1.1e−10
2.56 1.0e−10
1.83 1.3e−05
Orphanet_Augmented_2021=NUT midline carcinoma ORPHA:443167
2.35 1.4e−09
3.06 1.0e−10
2.15 1.2e−06
Running Enrichment Score
Ranked List Metric
Rank in Ordered Dataset

Figure S16. "Cancer-related" GSEA profiles in liver, adipose tissue and skeletal muscle from 5 month old *Mlx*KO mice relative to WT controls

Normalized enrichment scores and q values are indicated in the upper right corner of each profile. Data used to generate the ridge plots shown in Figure 6C are re-graphed and included here along with additional representative profiles. See File S3 for a complete list of all gene sets of significance included in this category.

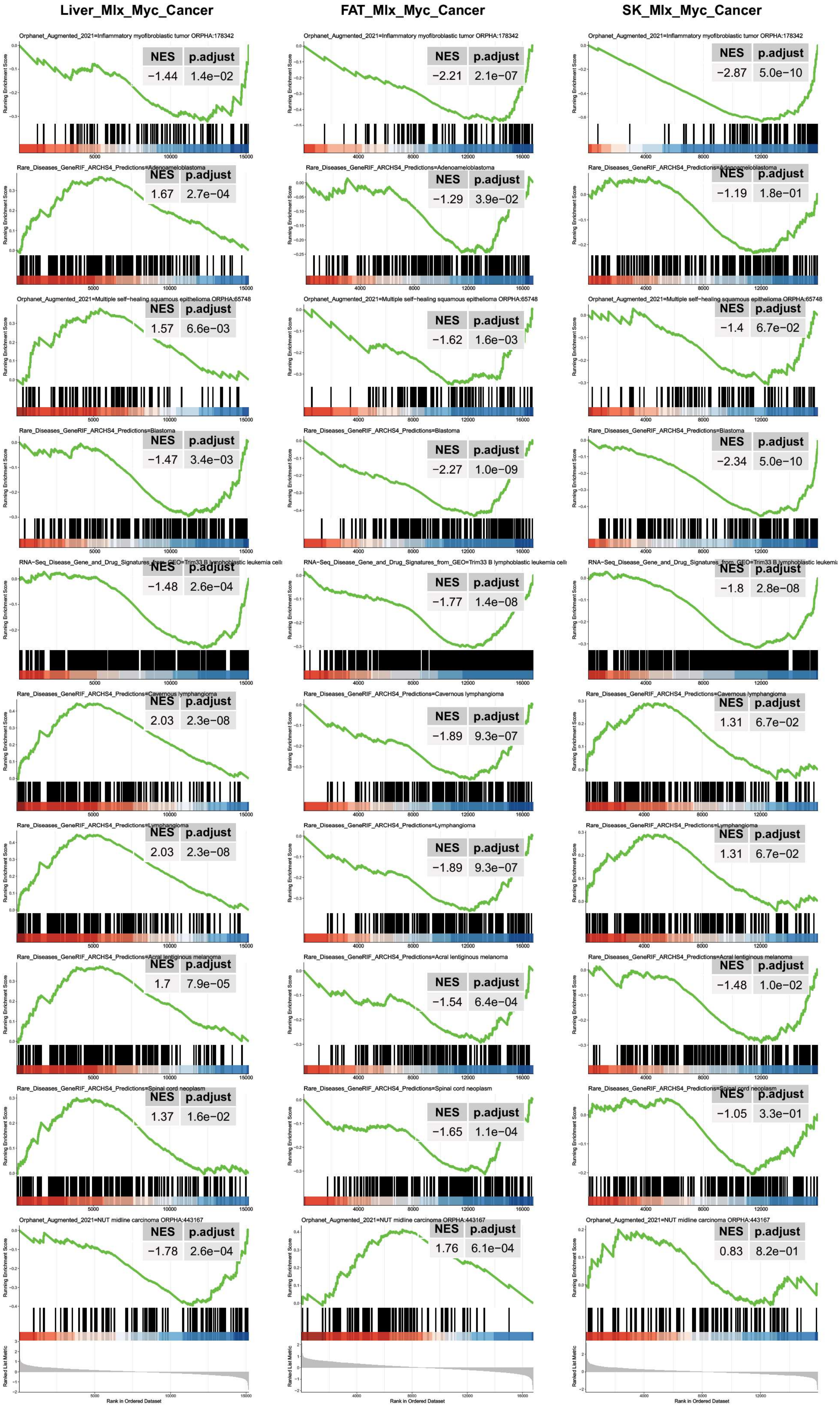
Liver_Mlx_Myc_Cancer
FAT_Mlx_Myc_Cancer
SK_Mlx_Myc_Cancer
Orphanet_Augmented_2021=Inflammatory myofibroblastic tumor ORPHA:178342
NES p.adjust
−1.44 1.4e−02
−2.21 2.1e−07
−2.87 5.0e−10
Rare_Diseases_GeneRIF_ARCHS4_Predictions=Adenoameloblastoma
1.67 2.7e−04
−1.29 3.9e−02
−1.19 1.8e−01
Orphanet_Augmented_2021=Multiple self−healing squamous epithelioma ORPHA:65748
1.57 6.6e−03
−1.62 1.6e−03
−1.4 6.7e−02
Rare_Diseases_GeneRIF_ARCHS4_Predictions=Blastoma
−1.47 3.4e−03
−2.27 1.0e−09
−2.34 5.0e−10
RNA−Seq_Disease_Gene_and_Drug_Signatures_from_GEO=Trim33 B lymphoblastic leukemia cells
−1.48 2.6e−04
−1.77 1.4e−08
−1.8 2.8e−08
Rare_Diseases_GeneRIF_ARCHS4_Predictions=Cavernous lymphangioma
2.03 2.3e−08
−1.89 9.3e−07
1.31 6.7e−02
Rare_Diseases_GeneRIF_ARCHS4_Predictions=Lymphangioma
2.03 2.3e−08
−1.89 9.3e−07
1.31 6.7e−02
Rare_Diseases_GeneRIF_ARCHS4_Predictions=Acral lentiginous melanoma
1.7 7.9e−05
−1.54 6.4e−04
−1.48 1.0e−02
Rare_Diseases_GeneRIF_ARCHS4_Predictions=Spinal cord neoplasm
1.37 1.6e−02
−1.65 1.1e−04
−1.05 3.3e−01
Orphanet_Augmented_2021=NUT midline carcinoma ORPHA:443167
−1.78 2.6e−04
1.76 6.1e−04
0.83 8.2e−01
Running Enrichment Score
Ranked List Metric
Rank in Ordered Dataset

Figure S17. "Cancer-related" GSEA profiles in liver, adipose tissue and skeletal muscle from 5 month old *Myc*KO mice relative to WT controls.
Normalized enrichment scores and q values are indicated in the upper right corner of each profile. Data used to generate the ridge plots shown in Figure 6C are re-graphed and included here along with additional representative profiles. See File S3 for a complete list of all gene sets of significance included in this category.

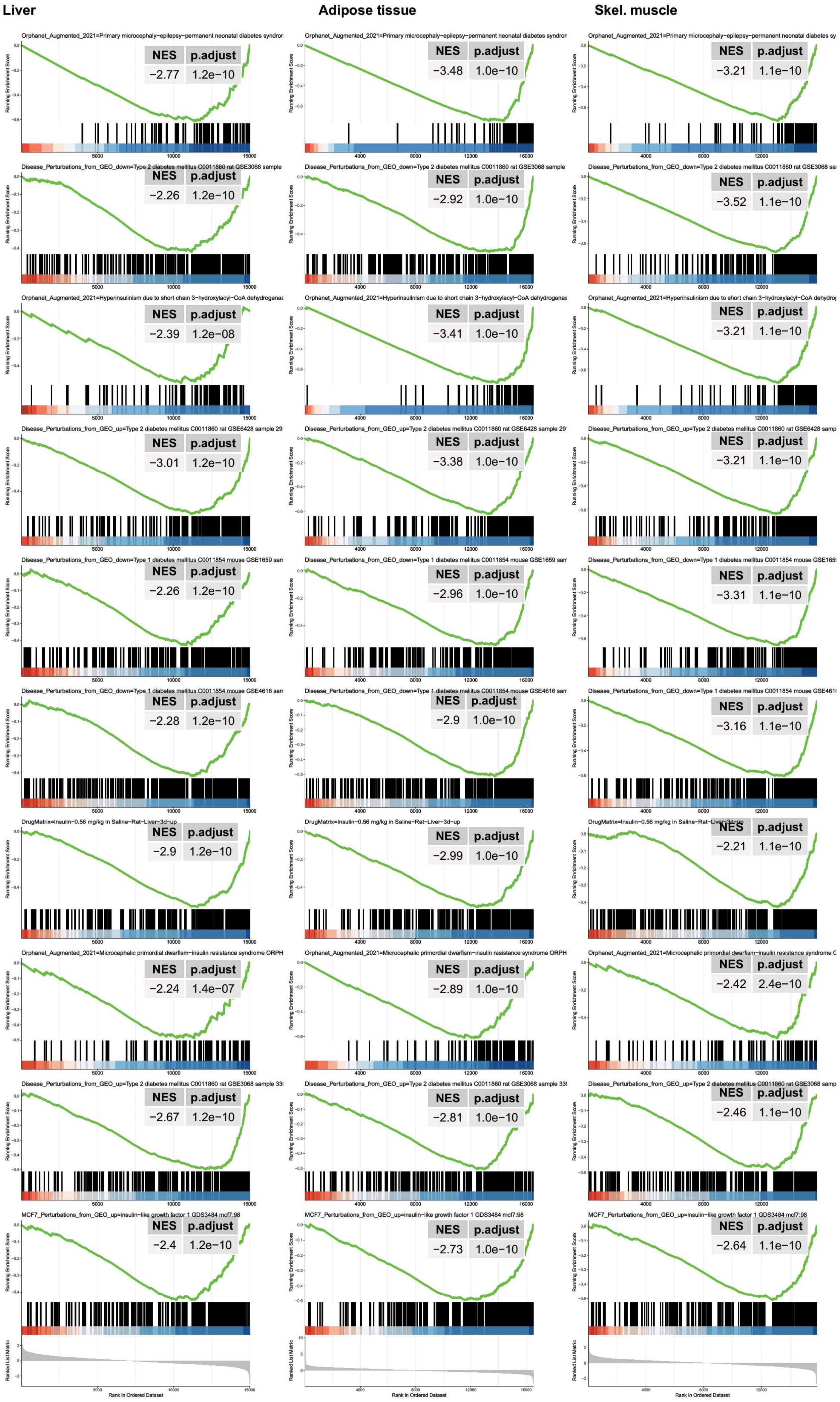
Liver
Adipose tissue
Skel. muscle
Orphanet_Augmented_2021=Primary microcephaly−epilepsy−permanent neonatal diabetes syndrom
NES p.adjust
−2.77 1.2e−10
Orphanet_Augmented_2021=Primary microcephaly−epilepsy−permanent neonatal diabetes syndrom
NES p.adjust
−3.48 1.0e−10
Orphanet_Augmented_2021=Primary microcephaly−epilepsy−permanent neonatal diabetes sy
NES p.adjust
−3.21 1.1e−10
Disease_Perturbations_from_GEO_down=Type 2 diabetes mellitus C0011860 rat GSE3068 sample
NES p.adjust
−2.26 1.2e−10
Disease_Perturbations_from_GEO_down=Type 2 diabetes mellitus C0011860 rat GSE3068 sample
NES p.adjust
−2.92 1.0e−10
Disease_Perturbations_from_GEO_down=Type 2 diabetes mellitus C0011860 rat GSE3068 sa
NES p.adjust
−3.52 1.1e−10
Orphanet_Augmented_2021=Hyperinsulinism due to short chain 3−hydroxylacyl−CoA dehydrogenas
NES p.adjust
−2.39 1.2e−08
Orphanet_Augmented_2021=Hyperinsulinism due to short chain 3−hydroxylacyl−CoA dehydrogenas
NES p.adjust
−3.41 1.0e−10
Orphanet_Augmented_2021=Hyperinsulinism due to short chain 3−hydroxylacyl−CoA dehydrog
NES p.adjust
−3.21 1.1e−10
Disease_Perturbations_from_GEO_up=Type 2 diabetes mellitus C0011860 rat GSE6428 sample 29
NES p.adjust
−3.01 1.2e−10
Disease_Perturbations_from_GEO_up=Type 2 diabetes mellitus C0011860 rat GSE6428 sample 29
NES p.adjust
−3.38 1.0e−10
Disease_Perturbations_from_GEO_up=Type 2 diabetes mellitus C0011860 rat GSE6428 samp
NES p.adjust
−3.21 1.1e−10
Disease_Perturbations_from_GEO_down=Type 1 diabetes mellitus C0011854 mouse GSE1659 sam
NES p.adjust
−2.26 1.2e−10
Disease_Perturbations_from_GEO_down=Type 1 diabetes mellitus C0011854 mouse GSE1659 sam
NES p.adjust
−2.96 1.0e−10
Disease_Perturbations_from_GEO_down=Type 1 diabetes mellitus C0011854 mouse GSE165
NES p.adjust
−3.31 1.1e−10
Disease_Perturbations_from_GEO_down=Type 1 diabetes mellitus C0011854 mouse GSE4616 sam
NES p.adjust
−2.28 1.2e−10
Disease_Perturbations_from_GEO_down=Type 1 diabetes mellitus C0011854 mouse GSE4616 sam
NES p.adjust
−2.9 1.0e−10
Disease_Perturbations_from_GEO_down=Type 1 diabetes mellitus C0011854 mouse GSE461
NES p.adjust
−3.16 1.1e−10
DrugMatrix=Insulin−0.56 mg/kg in Saline−Rat−Liver−3d−up
NES p.adjust
−2.9 1.2e−10
DrugMatrix=Insulin−0.56 mg/kg in Saline−Rat−Liver−3d−up
NES p.adjust
−2.99 1.0e−10
DrugMatrix=Insulin−0.56 mg/kg in Saline−Rat−Liver−3d−up
NES p.adjust
−2.21 1.1e−10
Orphanet_Augmented_2021=Microcephalic primordial dwarfism−insulin resistance syndrome ORPH
NES p.adjust
−2.24 1.4e−07
Orphanet_Augmented_2021=Microcephalic primordial dwarfism−insulin resistance syndrome ORPH
NES p.adjust
−2.89 1.0e−10
Orphanet_Augmented_2021=Microcephalic primordial dwarfism−insulin resistance syndrome C
NES p.adjust
−2.42 2.4e−10
Disease_Perturbations_from_GEO_up=Type 2 diabetes mellitus C0011860 rat GSE3068 sample 33
NES p.adjust
−2.67 1.2e−10
Disease_Perturbations_from_GEO_up=Type 2 diabetes mellitus C0011860 rat GSE3068 sample 33
NES p.adjust
−2.81 1.0e−10
Disease_Perturbations_from_GEO_up=Type 2 diabetes mellitus C0011860 rat GSE3068 samp
NES p.adjust
−2.46 1.1e−10
MCF7_Perturbations_from_GEO_up=insulin−like growth factor 1 GDS3484 mcf7:98
NES p.adjust
−2.4 1.2e−10
MCF7_Perturbations_from_GEO_up=insulin−like growth factor 1 GDS3484 mcf7:98
NES p.adjust
−2.73 1.0e−10
MCF7_Perturbations_from_GEO_up=insulin−like growth factor 1 GDS3484 mcf7:98
NES p.adjust
−2.64 1.1e−10
Running Enrichment Score
Ranked List Metric
Rank in Ordered Dataset

Figure S18. "T1D- and T2D-related" GSEA profiles in liver, adipose tissue and skeletal muscle from 5 month old *Mlx*KO mice relative to WT controls

Normalized enrichment scores and q values are indicated in the upper right corner of each profile. Data used to generate the ridge plots shown in Figure 6D are re-graphed and included here along with additional representative profiles. See File S4 for a complete list of all gene sets of significance included in this category.

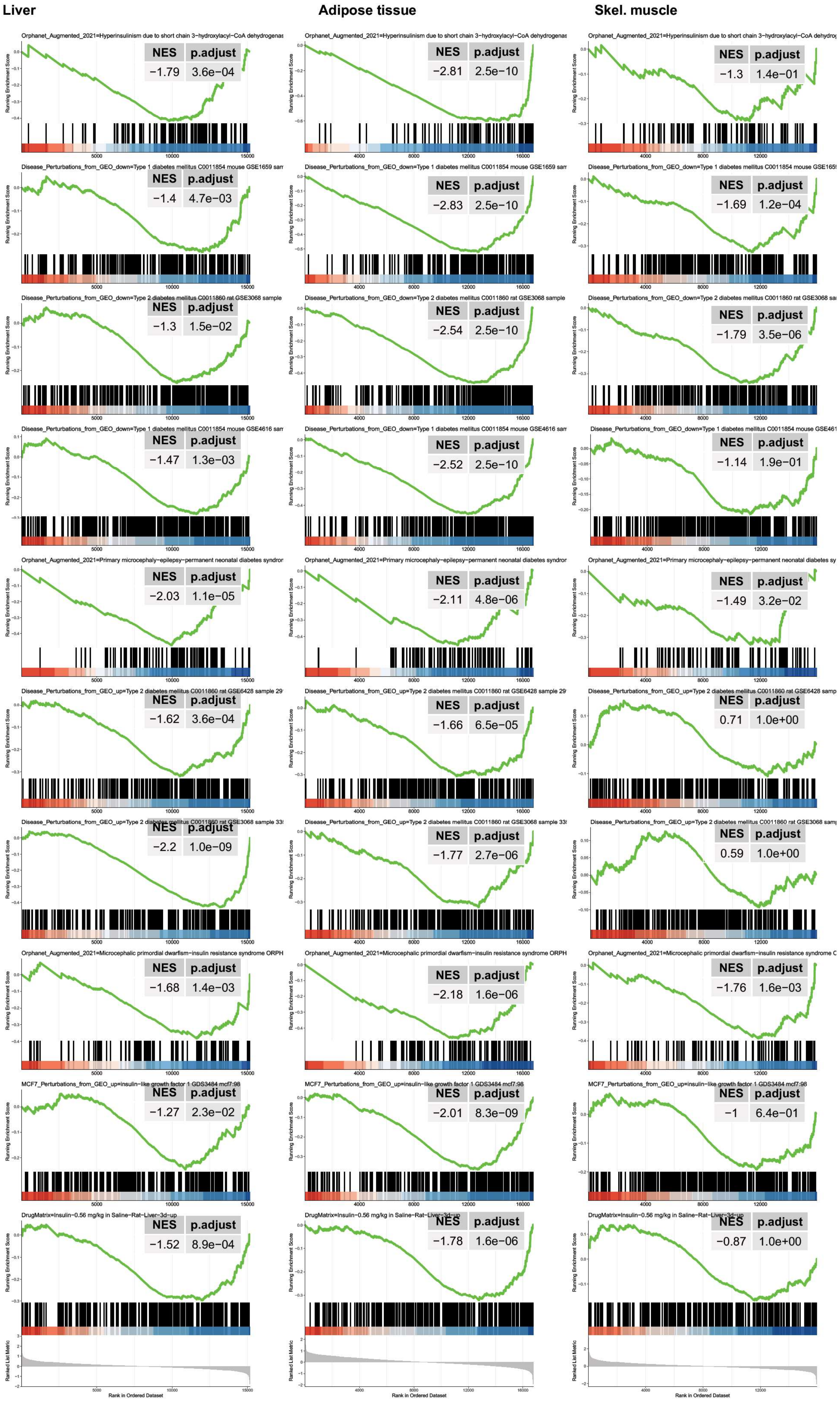

Liver
Adipose tissue
Skel. muscle
Orphanet_Augmented_2021=Hyperinsulinism due to short chain 3−hydroxylacyl−CoA dehydrogenas
NES p.adjust
−1.79 3.6e−04
−2.81 2.5e−10
−1.3 1.4e−01
Disease_Perturbations_from_GEO_down=Type 1 diabetes mellitus C0011854 mouse GSE1659 sam
NES p.adjust
−1.4 4.7e−03
−2.83 2.5e−10
−1.69 1.2e−04
Disease_Perturbations_from_GEO_down=Type 2 diabetes mellitus C0011860 rat GSE3068 sample
NES p.adjust
−1.3 1.5e−02
−2.54 2.5e−10
−1.79 3.5e−06
Disease_Perturbations_from_GEO_down=Type 1 diabetes mellitus C0011854 mouse GSE4616 sam
NES p.adjust
−1.47 1.3e−03
−2.52 2.5e−10
−1.14 1.9e−01
Orphanet_Augmented_2021=Primary microcephaly−epilepsy−permanent neonatal diabetes syndror
NES p.adjust
−2.03 1.1e−05
−2.11 4.8e−06
−1.49 3.2e−02
Disease_Perturbations_from_GEO_up=Type 2 diabetes mellitus C0011860 rat GSE6428 sample 29
NES p.adjust
−1.62 3.6e−04
−1.66 6.5e−05
0.71 1.0e+00
Disease_Perturbations_from_GEO_up=Type 2 diabetes mellitus C0011860 rat GSE3068 sample 33
NES p.adjust
−2.2 1.0e−09
−1.77 2.7e−06
0.59 1.0e+00
Orphanet_Augmented_2021=Microcephalic primordial dwarfism−insulin resistance syndrome ORPH
NES p.adjust
−1.68 1.4e−03
−2.18 1.6e−06
−1.76 1.6e−03
MCF7_Perturbations_from_GEO_up=insulin−like growth factor 1 GDS3484 mcf7:98
NES p.adjust
−1.27 2.3e−02
−2.01 8.3e−09
−1 6.4e−01
DrugMatrix=Insulin−0.56 mg/kg in Saline−Rat−Liver−3d−up
NES p.adjust
−1.52 8.9e−04
−1.78 1.6e−06
−0.87 1.0e+00
Running Enrichment Score
Ranked List Metric
Rank in Ordered Dataset

Figure S19. "T1D- and T2D-related" GSEA profiles in liver, adipose tissue and skeletal muscle from 5 month old *Myc*KO mice relative to WT controls [7e].

Normalized enrichment scores and q values are indicated in the upper right corner of each profile. Data used to generate the ridge plots shown in Figure 6D are re-graphed and included here along with additional representative profiles. See File S4 for a complete list of all gene sets of significance included in this category.

Other Supporting Information for this manuscript include the following:

File S1. Complete list of all gene sets of significance from which the GSEA profiles depicted in Figure 6A and Figures S3-S9 are drawn.

File S2. Complete list of all gene sets of significance from which the GSEA profiles depicted in Figure 6B and Figures S10-S15.

File S3. **Complete list of all "Cancer-related" gene sets of significance.** Within this category are those sets depicted in Figure 6C and Figure S16-S17.

File S4. Complete list of all T1D- and T2D-related gene sets of significance. Within this category are those sets depicted in Figure 6D and Figure S18-S19.

File S5. Complete list of all gene sets used to generate the heat map shown in Figure 7A.

File S6. Identities of the 3191 gene sets pertaining to the immune response, inflammation and cytokine production depicted in Figure 7B.

File S7. Detailed information about the RNA-seq datasets used in Figure 7C-D. These datasets were obtained from GEO databases and include data from normal mice of various strains, which were maintained on either standard or high-fat diets (HFDs).
